# Advanced Cancer Starvation Therapy by Simultaneous Deprivation of Lactate and Glucose Using a MOF Nanoplatform

**DOI:** 10.1002/advs.202101467

**Published:** 2021-08-07

**Authors:** Jiantao Yu, Zixiang Wei, Qing Li, Feiyan Wan, Zhicong Chao, Xindan Zhang, Li Lin, Hong Meng, Leilei Tian

**Affiliations:** ^1^ School of Advanced Materials Peking University Shenzhen Graduate School Peking University Shenzhen 518055 China; ^2^ Department of Materials Science and Engineering Southern University of Science and Technology 1088 Xueyuan Blvd., Nanshan District Shenzhen Guangdong 518055 P. R. China

**Keywords:** glucose, lactate, metal‐organic frameworks, monocarboxylate transporter 1, starvation therapy

## Abstract

Recent investigations reveal that lactate is not a waste product but a major energy source for cells, especially in the mitochondria, which can support cellular survival under glucose shortage. Accordingly, the new understanding of lactate prompts to target it together with glucose to pursue a more efficient cancer starvation therapy. Herein, zeolitic imidazolate framework‐8 (ZIF‐8) nanoplatforms are used to co‐deliver *α*‐cyano‐4‐hydroxycinnamate (CHC) and glucose oxidase (GOx) and fulfill the task of simultaneous depriving of lactate and glucose, resulting in a new nanomedicine CHC/GOx@ZIF‐8. The synthesis conditions are carefully optimized in order to yield monodisperse and uniform nanomedicines, which will ensure reliable and steady therapeutic properties. Compared with the strategies aiming at a single carbon source, improved starvation therapy efficacy is observed. Besides, more than boosting the energy shortage, CHC/GOx@ZIF‐8 can block the lactate‐fueled respiration and relieve solid tumor hypoxia, which will enhance GOx catalysis activity, depleting extra glucose, and producing more cytotoxic H_2_O_2_. By the synergistically enhanced anti‐tumor effect, both in vitro and in vivo cancer‐killing efficacies of CHC/GOx@ZIF‐8 show twice enhancements than the GOx mediated therapy. The results demonstrate that the dual‐depriving of lactate and glucose is a more advanced strategy for strengthening cancer starvation therapy.

## Graphical abstract

Metal‐organic‐framework nanomedicines encapsulating an inhibitor of monocarboxylate transporters 1 and glucose oxidase are developed for efficient glucose depletion while inhibiting the lactate influx. At meanwhile, inhibiting the lactate influx can improve the intracellular oxygen pressure, which subsequently promotes the catalytic activity of glucose oxidase. Cancer cells are substantially killed from ATP shortage and high ROS levels.

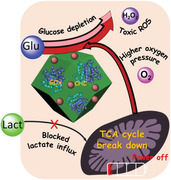

## 1 Introduction

Cancer cells are greedy for glucose as energy to drive their rapid proliferation. Accordingly, cancer starvation therapy based on glucose‐deprivation is emerging as an effective method for suppressing tumor growth and survival.^[^ ^1^ ^]^ In most of these researches, glucose oxidase (GOx) was applied to deplete glucose by converting to gluconic acid and hydrogen peroxide (H_2_O_2_).^[^ ^2^ ^]^ The overproduced H_2_O_2_ will induce systemic toxicity, which will enhance the cancer‐killing efficiency in addition to glucose‐blocking induced energy shortage. However, this process will consume O_2_ and elevate the local hypoxia in vivo. And considering the inherent hypoxic conditions in the solid tumor, the efficiency of GOx‐mediated cancer starvation therapy was restrained by the insufficient O_2_ supply.^[^ ^3^ ^]^ Therefore, many efforts have been made to improve the efficacy of GOx‐mediated starvation efficacy. For example, catalysts such as MnO_2_^[^ ^4^ ^]^ and catalase^[^ ^5^ ^]^ were used to convert H_2_O_2_ to H_2_O and O_2_ to improve the local oxygen pressure, which could subsequently promote the catalytic activity of GOx. Unfortunately, for this strategy, the improved oxygen pressure was at the price of sacrificing the toxic H_2_O_2_, which will make the treatment less effective. On the other hand, substrates such as lactate,^[^ ^6^ ^]^ glutamine,^[^ ^7^ ^]^ and aspartate^[^ ^8^ ^]^ may compensate for the starvation of glucose, subsequently pulling down the efficacy of starvation therapy.

The glucose consumed by cancer cells further metabolizes predominantly through glycolysis, thus producing high levels of lactate even in oxygen‐rich conditions (Warburg Effect).^[^ ^9^ ^]^ Lactate was previously considered a waste product that must be eliminated by cancer cells despite the fact that blood lactate is a prominent fuel for mitochondrial metabolism (tricarboxylic acid cycle, TCA cycle) in both normal and cancer tissues.^[^ ^6^, ^10^ ^]^ Oxidative cancer cells can directly use lactate in the tumor microenvironment (TME) to support the aggressive development of tumors and preserve cellular survival under glucose shortage.^[^ ^6a^ ^]^ Some recent findings revealed that lactate would support cancer angiogenesis and metastasis,^[^ ^11^ ^]^ acts as a catalyst to activate mutated genes into cancer,^[^ ^12^ ^]^ and interferes with the immune response to cancer.^[^ ^13^ ^]^ The essential roles of lactate, such as the major signaling molecule and the regulator of cancer development, have been realized gradually and, very recently, applied in nanomedicine design. For instance, direct lactate removal using lactate oxidase was used to regulate the lactate abundance in TME, which subsequently generated toxic H_2_O_2_ to kill cells.^[^ ^14^ ^]^ Also, approaches targeting the dominating enzyme of pyruvate/lactate shuttle, lactate dehydrogenase, were used to inhibit the lactate generation in TME.^[^ ^15^ ^]^ Monocarboxylate transporters (MCTs) are the guards of lactate flux between cells and the microenvironments. MCT1 inhibitory strategies, including using inhibitors and gene regulations, were reported to interfere with lactate‐fueled respiration in mitochondria.^[^ ^6^, ^12^, ^16^ ^]^ Blocking the lactate influx of cancer cells could improve the local oxygen pressure, which subsequently improves the therapeutic efficacy of oxygen‐consuming therapeutics, including photodynamic therapy^[^ ^16^ ^]^ and radiotherapy.^[^ ^17^ ^]^

Besides these essential roles, lactate is also the major energy source for the cell in addition to glucose; therefore, the influence of lactate blocking on GOx mediated cancer starvation therapy is attractive to be explored. Herein, a new strategy of starvation therapy by dual‐blocking the main energy sources, glucose and lactate, simultaneously, has been designed, in which glucose will be depleted by GOx catalysis, and the cellular influx of exogenous lactate is blocked by MCT1 inhibitor, *α*‐cyano‐4‐hydroxycinnamate (CHC). To co‐deliver CHC and GOx, the porous and biocompatible zeolitic imidazolate framework‐8 (ZIF‐8) was selected as the carrying‐nanoplatform, which has been widely applied in the delivery of biomacromolecules, such as protein^[^ ^18^ ^]^ and DNA,^[^ ^19^ ^]^ as well as small‐molecular drugs.^[^ ^20^ ^]^ Moreover, ZIF‐8 can sensitively decompose and release the cargos under weak acidic environments, which will promise the targeting delivery of drugs toward cancers.^[^ ^21^ ^]^ Benefiting from the strong interaction between Zn^2+^ and carboxyl groups in CHC and GOx, we successfully realized the in situ co‐loading of CHC and GOx during the crystallization process of ZIF‐8 to obtain the final product, CHC/GOx@ZIF. In this structure, both GOx and CHC were entrapped in the crystal structures of ZIF‐8 to rescue from the burst release of the cargos to normal tissues. Moreover, we demonstrated that *N*,*N*‐dimethylformamide (DMF) played important roles in CHC/GOx@ZIF‐8 synthesis, including weak basic property, structure‐directing, and excellent dissolving ability toward CHC and Zn^2+^. The as‐obtained monodisperse and uniform nanomedicines will ensure reliable and steady therapeutic properties.

**Scheme 1:**
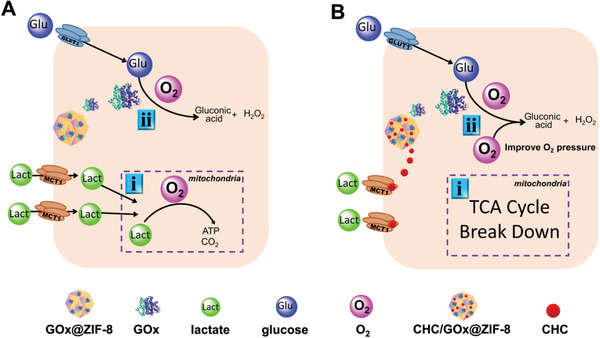
The intracellular therapeutic mechanisms of A) GOx mediated cancer starvation therapy and B) dual‐depriving cancer starvation therapy (glucose is depleted by GOx and the cellular influx of exogenous lactate is blocked by MCT1 inhibitor, CHC). Both therapies are conducted by the ZIF‐8 nanoplatforms.

Through the careful cellular and in vivo experiments, we demonstrated that both lactate and glucose could fuel cell growth, and the simultaneous depriving of lactate and glucose could reinforce the effect of the starvation therapy. Moreover, the lactate‐fueled respiration process is an oxygen consumption process in addition to generating ATP and producing energy. Therefore, utilizing CHC to inhibit MCT1 mediated lactate influx can efficiently relieve the hypoxia condition of tumor cells, which consequently promoted the catalytical activity of GOx, leading to further glucose depletion and toxic H_2_O_2_ production (**Scheme** **1**). As a result, an apoptosis up to 71.7% was observed for the group treated with CHC/GOx@ZIF‐8 in comparison with 44.3% cell‐apoptosis of the group treated with GOx@ZIF‐8. Also, the in vivo tumor growth inhibitory effect of CHC/GOx@ZIF‐8 was found twice‐enhanced in comparison with GOx@ZIF‐8, demonstrating the synergistically enhanced anti‐tumor effects of the dual‐depriving strategy.

## 2 Results and Discussion

### 2.1 Blocking the Influx of Exogenous Lactate is Necessary to Improve the Efficiency of Starvation Therapy

**Figure 1:**
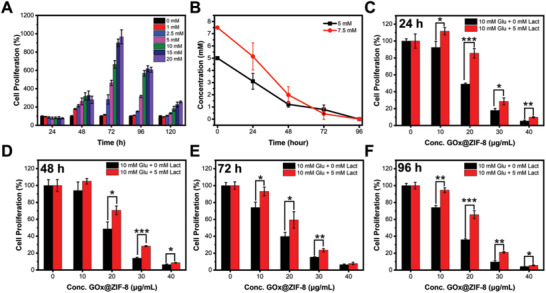
Lactate can preserve cellular survival under glucose starvation. A) Cell proliferation after incubation with different concentrations of lactate for different periods. B) Lactate concentration of the cell culture medium after different incubation periods. C–F) Cell proliferation after incubation with different concentrations of GOx@ZIF‐8 for 24, 48, 72, and 96 h in media containing glucose (10 mm) or glucose (10 mm) + lactate (5 mm). Data are presented as mean ± SD, *n* = 3, *p < 0.05, **p < 0.01, and ***p < 0.001. Cells without any treatment were set as the control group and cell proliferation of the control group was set as 100%.

GOx induced glucose‐depletion has become the most used strategy for cancer starvation therapy. However, some recent reports revealed that lactate was also an essential energy source as exogenous lactate transported by MCT1 could serve as the TCA cycle substrate to fuel the growth of cancer cells directly.^[^ ^6^, ^10^, ^22^ ^]^ In order to verify the role of exogenous lactate in supporting the survival of tumor cells after the depletion of glucose, MCF‐7 cells (expressing MCT1) were cultured in the media supplied with different concentrations of lactate but free of glucose, glutamine, and pyruvate (**Figure** **1**A). The pH values of all the media were balanced to 7.4 by using sodium lactate and lactate acid to eliminate the influence from pH. When the incubation time exceeded 48 h, cell proliferation increased by a wide margin for the conditions with lactate supplies. At the incubation time of 48, 72, 96, and 120 h, the groups incubated with 10 mm of lactate, which is comparable to the lactate concentration in solid tumors, showed cell proliferation 3.1, 5.6, 6.6, and 1.8‐fold of the group without lactate supply. Moreover, significant lactate consumptions in the media were observed in a time‐dependent manner (Figure 1B), which indicated that the exogenous lactate could participate in cell metabolism and support cell survival. Accordingly, taking glucose as the only nutrition, we found that cells were efficiently starved to death under the treatment of GOx@ZIF‐8. Considering the abundant existence of lactate in the TME, cocktails of glucose (10 mm) and lactate (5 mm) were exogenously added to the cell culture media under GOx@ZIF‐8 treatment (Figure 1C–F), which resulted in pronouncedly increased cell survivals with the energy support of lactate. The result is consistent with the findings that the intracellular lactate uptake will fuel TCA cycle for ATP generation. Thus, we assumed that blocking the influx of lactate would be necessary to improve the efficiency of GOx mediated starvation therapy.

### 2.2 Optimization of the Morphology of Co‐Crystallized CHC@ZIF‐8

**Figure 2:**
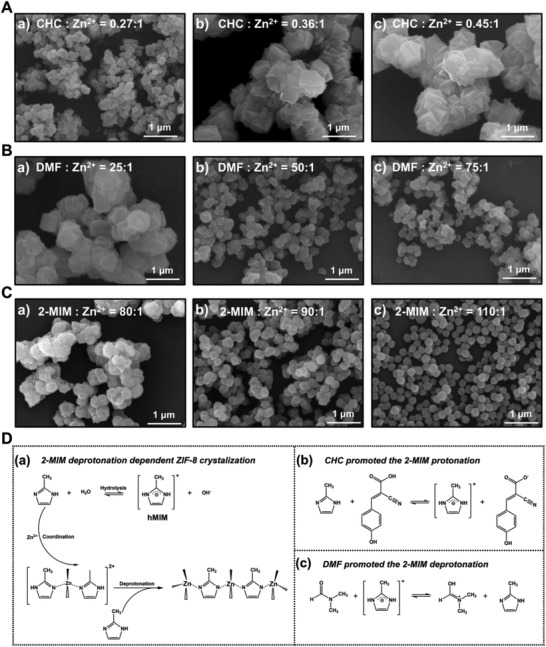
Fine‐tune the crystal morphology of CHC@ZIF‐8 by increasing the usage of DMF and 2‐MIM. SEM images of A) CHC@ZIF‐8 crystals synthesized at Zn^2+^:2‐MIM:H_2_O molar ratio of 1:70:1245 with increasing dosages of CHC from a) to c). B) CHC@ZIF‐8 crystals synthesized at Zn^2+^:CHC:2‐MIM:H_2_O molar ratio of 1:0.36:70:1245 with increasing dosages of DMF from a) to c) and C) CHC@ZIF‐8 crystals synthesized at Zn^2+^:CHC:H_2_O:DMF molar ratio of 1:0.36:1245:50 with increasing dosages of 2‐MIM from a) to c). D) Illustration of the a) crystallization process of ZIF‐8 crystals in aqueous, b) strengthened protonation of 2‐MIM by CHC, and c) promoted deprotonation of 2‐MIM by DMF. Scale bar: 1 µm.

Physical absorption is widely used for loading cargos that have smaller sizes than the pore sizes of ZIF‐8 nanoparticles. However, this method suffers from the easy desorption and burst release of the cargos. Therefore, we tried to load CHC, an efficient MCT1 inhibitor, through the co‐crystallization method to enhance the delivery system's stability. By this method, CHC was dissolved together with 2‐methylimidazole (2‐MIM) by water, and crystals were formed with the injection of Zn^2+^. As CHC shows good interactions with both Zn^2+^ and 2‐MIM through the carboxyl group, it could be efficiently encapsulated in situ during ZIF‐8 crystallization. According to the electronic microscopy characterization, the crystal size of CHC@ZIF‐8 was much larger than that of ZIF‐8 synthesized at the same conditions (**Figure** **2**A; Figure S1, Supporting Information). To clarify the influence of CHC on the morphology of CHC@ZIF‐8, we fixed the molar ratio of Zn^2+^:2‐MIM:water to 1:70:1245 while altered the molar ratio of CHC:Zn^2+^ to synthesize CHC@ZIF‐8 crystals. We found that the crystals turned to aggregate and intergrew to form irregular particles as the usage of CHC increased (Figure 2A). The acidity of CHC would hinder the deprotonation process of 2‐MIM, which would subsequently slow down the crystallization growth of ZIF‐8 and result in the formation of larger nanocrystals. As increasing the ligand concentration can accelerate the deprotonation of 2‐MIM,^[^ ^23^ ^]^ we attempted to adjust the crystal size of CHC@ZIF‐8 by increasing the usage of 2‐MIM. Unfortunately, the particles were still aggregated and irregular even the 2‐MIM concentration was near‐saturated (Figure S2, Supporting Information), which indicated the strong interaction between CHC and 2‐MIM. Thus, we assumed that the formation of irregular particles might be caused by the following reasons. 1) The nucleation of ZIF‐8 nanocrystal is a process accompanied by the deprotonation of the 2‐MIM ligands, which would be hindered by the acidity of CHC (Figure 2D); 2) CHC decreased the solubility of the formed ZIF‐8 crystals. Accordingly, a co‐solvent is required to enhance the solubility of the hydrophobic CHC in the composite and accelerate the crystallization of ZIF‐8. In addition to the good dissolving ability of both CHC and Zn^2+^, DMF was selected as the co‐solvent for its weak alkaline (Figure 2D) and structure‐directing effect for MOF crystallization.^[^ ^24^ ^]^ As shown in Figure 2B, the introduction of DMF significantly decreased the crystal size of CHC@ZIF‐8. However, the formed CHC@ZIF‐8 crystals still tended to aggregate even the molar ratio of DMF:Zn^2+^ reached 75:1. For the consideration of protecting the activity of GOx in the co‐loading process with CHC, we increased the usage of 2‐MIM instead of further increasing the usage of DMF to decrease the CHC@ZIF‐8 crystals. Finally, the molar ratio of Zn^2+^:2‐MIM:CHC:water:DMF was optimized to 1:110:0.36:1245:50 (Figure 2C; Figure S3, Supporting Information). Under this condition, the aggregation and the intergrowing problem have been solved, and isolated nanocrystals with diameters of 177.6 ± 19.5 nm could be synthesized (Figure S4, Supporting Information). For this optimized ratio, if DMF is replaced by TEA (a basic reagent), methanol or ethanol (the reported structure‐directing agents), and DMSO (a good solvent of CHC), the resulted composite nanocrystals still showed severe aggregations (Figure S5, Supporting Information). Although TEA could adjust the crystal growth process to a certain degree as it could neutralize the acidity of CHC and promote the deprotonation of 2‐MIM,^[^ ^25^ ^]^ it lacks the structure‐directing effect toward the ZIF‐8 growth and good solubility toward CHC. Thus, the final products still showed unsatisfactory morphology. These results further demonstrated the triple effects of DMF, including weak basic property, structure‐directing, and excellent dissolving ability toward CHC and Zn^2+^, played important roles in CHC@ZIF‐8 synthesis.

### 2.3 Co‐Loading of CHC and GOx by ZIF‐8 Crystals

**Figure 3:**
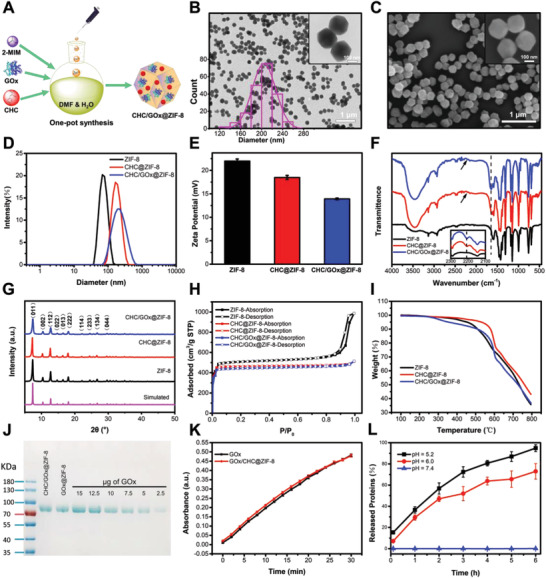
Synthesis and characterization of CHC/GOx@ZIF‐8 crystals. A) Illustration of the one‐pot synthesis of CHC/GOx@ZIF‐8. B) TEM images with size distribution and C) SEM images of CHC/GOx@ZIF‐8 crystals. D) Hydrodynamic size distribution, E) zeta potential, F) FT‐IR absorption spectra, G) PXRD, H) BET, and I) TGA absorption spectra of ZIF‐8, CHC@ZIF‐8, and CHC/GOx@ZIF‐8. J) SDS‐PAGE analysis of GOx loading efficiency in GOx@ZIF‐8 and CHC/GOx@ZIF‐8. K) Kinetics of TMB oxidation catalyzed by free GOx and GOx released from CHC/GOx@ZIF‐8. L) The pH‐dependent release profile of CHC/GOx@ZIF‐8. Scale bar in Figure 3B,C: 1 µm. Scale bar in the inserts of Figure 3B,C: 100 nm.

Based on the optimized fabrication conditions for CHC@ZIF‐8, we further synthesized CHC/GOx@ZIF‐8 by one‐pot loading CHC and GOx in ZIF‐8 (**Figure** **3**A). The weight ratio between CHC and GOx was determined by the optimized synergistic anti‐tumor effect (Figure S6, Supporting Information). Uniform nano‐crystals with an average diameter of 204.5 ± 22.6 nm were obtained as characterized by TEM and SEM (Figure 3B,C), which was larger than CHC@ZIF‐8 (177.6 ± 19.5 nm) (Figure S4, Supporting Information). Due to cargos incorporation, from ZIF‐8, CHC@ZIF‐8, to CHC/GOx@ZIF‐8, we observed that the particle sizes gradually increased (by dynamic light scattering analysis, Figure 3D), and zeta potentials decreased as both CHC and GOx are negatively charged under the physiological condition (Figure 3E). For FT‐IR spectral analysis (Figure 3F), characteristic peaks at 2210 and 1650 cm^−1^, corresponding to the cyano groups stretching vibration in CHC and the vibration of peptide‐bond in GOx were observed in CHC/GOx@ZIF‐8. Powder X‐ray diffraction (PXRD) analysis disclosed that the incorporation of GOx and CHC would not change the crystallinity of ZIF‐8 (Figure 3G). According to the BET analysis (Figure 3H), all three samples displayed type‐I adsorption‐desorption isotherms. The BET surface and pore volume of CHC@ZIF‐8 decreased to 1397.0 m^2^ g^−1^ and 0.79 cm^3^ g^−1^ in comparison with that of ZIF‐8 (1550.7 m^2^ g^−1^ and 1.55 cm^3^ g^−1^), as CHC was filled in the pores due to its small size. Differently, CHC/GOx@ZIF‐8 showed comparable BET surface and pore volume (1345.0 m^2^ g^−1^ and 0.80 cm^3^ g^−1^) to CHC@ZIF‐8, which indicated that GOx, a biomacromolecule, was directly encapsulated into the crystal of ZIF‐8. Further characterized by TGA under N_2_ atmosphere, all the three samples showed good thermal stabilities with small weight loss (<10%) up to 500 °C (Figure 3I). The loading capacities of CHC in CHC@ZIF‐8 and CHC/GOx@ZIF‐8 were 2.6 and 2.5 wt% according to the measurement by a UV–vis spectrophotometer (Figure S7, Supporting Information). The loading capacities of GOx in GOx@ZIF‐8 and CHC/GOx@ZIF‐8 were calculated to be 11.5 and 11.9 wt%, according to the SDS‐PAGE analysis (Figure 3J). The SDS‐PAGE analysis also confirmed the structural integrity of GOx after the encapsulation. Further, the catalytic activity of GOx was evaluated by the horseradish peroxidase/3,3′,5,5′‐tetramethylbenzidine (HRP/TMB)‐based colorimetric assay (Figure 3K). Compared with the native GOx, GOx in CHC/GOx@ZIF‐8 was found to maintain its catalytic activity after encapsulation. Finally, the cellular internalization of CHC/GOx@ZIF‐8 was characterized by laser scanning confocal microscope (LSCM) and flow cytometry (FCM). Efficient cellular internalization was observed after a 20‐min incubation (Figure S8, Supporting Information). In order to detect the release of GOx from CHC/GOx@ZIF‐8 under the acid TME, the fluorophore‐labeled CHC/GOx@ZIF‐8 samples were incubated in PBS of different pH values (5.2, 6.0, and 7.4) for different periods. As shown in Figure 3L, the release amount of GOx was negligible at pH = 7.4, while efficient releases were observed under the acidic conditions (pH = 5.2 and 6.0). Approximately 50% of GOx would be released after a 3‐h incubation at pH 6.0. Therefore, the efficient intracellular delivery and cargo release make CHC/GOx@ZIF‐8 a tumor‐targeting and accumulating nanomedicine.

### 2.4 Efficient Cancer Starvation Therapy by CHC/GOx@ZIF‐8

**Figure 4:**
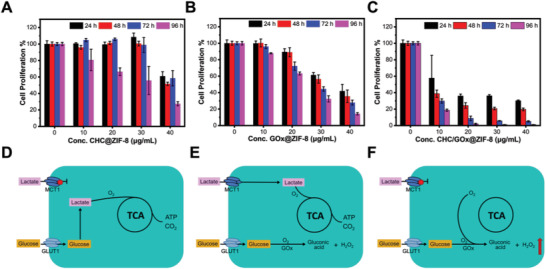
A–C) Cell proliferation and D–F) metabolism profiles of cells incubated with CHC@ZIF‐8 (A,D), GOx@ZIF‐8 (B,E), and CHC/GOx@ZIF‐8 (C,F) in medium containing glucose (10 mm) and lactate (10 mm). Data are presented as mean ± SD, *n* = 3. Cells without any treatment were set as the control group and cell proliferation of the control group was set as 100%.

Cancer cells expressing MCT1 could directly use exogenous lactate in addition to glucose to feed the TCA cycle for ATP production (Figure 1). Consequently, the reinforcing effect of blocking the MCT1‐mediated influx of exogenous lactate on starvation therapy was further explored (Scheme 1). First, MCT1‐expressing MCF‐7 cells were maintained in DMEM medium supplied with 10 mm of lactate as the only nutrition and incubated with different concentrations of CHC. According to the cell proliferation test by cell counting kit‐8 (CCK‐8; Figure S9, Supporting Information), CHC with a concentration down to 10 µm could efficiently inhibit the lactate‐fueled cell growth. And as expected, if glucose was supplied as the only nutrition, cell proliferation would not change at the presence of CHC (the concentration up to 2.5 mm) (Figure S10, Supporting Information), which indicated the good specificity of CHC for inhibiting the influx of exogenous lactate. Like CHC, CHC@ZIF‐8 could also significantly inhibit the growth of the cells incubated in the medium supplied with 10 mm lactate (Figure S11, Supporting Information). Next, we cultured cells in media supplied with 10 mm lactate and 10 mm glucose to mimic the tumor environment in which exogenous lactate and glucose co‐exist and tested the cell‐killing properties of the nano‐crystals.^[^ ^6a^ ^]^ Biocompatibility of ZIF‐8 crystals was first investigated in media supplied with the dual carbon sources of lactate and glucose or a single carbon source of them, and no detectable cytotoxicity was observed when cells were incubated with 40 µg mL^−1^ of ZIF‐8 crystals for 96 h (Figures S12,S13, Supporting Information). Under the same condition, the cell‐killing capabilities of CHC@ZIF‐8 (**Figure** **4**A), GOx@ZIF‐8 (Figure 4B), and CHC/GOx@ZIF‐8 (Figure 4C) were compared and analyzed. CHC/GOx@ZIF‐8 exhibited a notably improved cell‐killing efficiency in comparison with CHC@ZIF‐8 and GOx@ZIF‐8. Especially at the low concentration of 10 µg mL^−1^, CHC/GOx@ZIF‐8 already exhibited considerable cytotoxicity, while the cytotoxicity of both CHC@ZIF‐8 and GOx@ZIF‐8 could be negligible. On one side, when glucose was eliminated by GOx@ZIF‐8, energy for cell survival could be compensated from MCT1 mediated lactate influx, which has been sufficiently proved by the previous results (Figures 1,4E). On the other side, if lactate influx was blocked by CHC@ZIF‐8, energy for cell survival could be supplied from the glucose transporter 1 mediated glucose influx (Figure 4D). The results proved our hypothesis that, as both lactate and glucose could fuel cell growth, the simultaneous depriving of lactate and glucose could reinforce the effect of the starvation therapy. Whereas it's interesting and worth noting that, rather than simple addition, a synergistic enhancement in therapeutic efficiency by the dual‐depriving strategy can be clearly observed. The interest in revealing the reasons stimulated us to conduct further investigations.

### 2.5 Mechanism of the Improved Starvation Therapy by CHC/GOx@ZIF‐8

**Figure 5:**
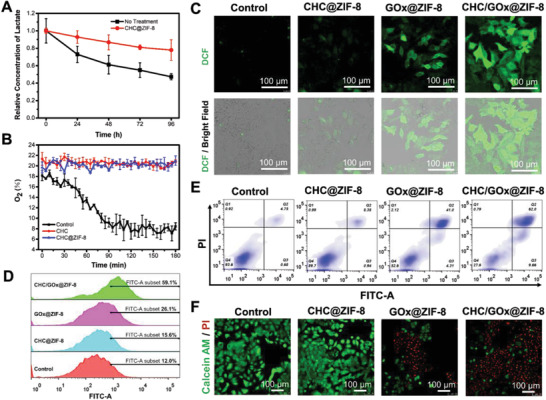
A) Lactate and B) O_2_ consumption profile of cells after different treatment. C) LSCM images and D) FCM analysis of cells stained with DCFH‐DA after incubation with CHC@ZIF‐8, GOx@ZIF‐8, and CHC/GOx@ZIF‐8 for 6 h. E) FCM analysis of cellular apoptosis and F) LSCM images of live/dead cells stained with calcein‐AM (green)/PI (red) after incubation with CHC@ZIF‐8, GOx@ZIF‐8, and CHC/GOx@ZIF‐8 for 24 h. Cell culturing media for this part are DMEM with 10% FBS, glucose (10 mm), and lactate (10 mm). Scale bar in Figure 5C,F: 100 µm.

Hypoxia, a common hallmark in all solid tumors, greatly correlates with tumor aggressiveness and poor prognosis, which also causes the resistance of various tumor treatments, including the GOx‐mediated cancer starvation therapyST. The glucose depletion efficiency was restrained by the hypoxic TME in vivo because O_2_ is necessary for the catalytical reaction of GOx. As we have proved that CHC and CHC@ZIF‐8 could block the lactate‐fueled respiration, this process's influence on the TME was studied. During a 3‐h incubation of MCF‐7 cells in the presence of CHC or CHC@ZIF‐8, the composition of the culture medium was monitored. Along with the restriction of lactate influx (**Figure** **5**A), we found that the oxygen consumption rate was also suppressed (Figure 5B) and the intracellular hypoxia condition was relieved (Figure S14, Supporting Information). This finding is interesting, which is reasonable as the lactate‐fueled respiration process is also an oxygen consumption process in addition to generating ATP and producing energy. Therefore, utilizing CHC to inhibit MCT1 mediated lactate influx will bring two beneficial outcomings to cancer therapy. 1) It can efficiently block the lactate‐fueled respiration process and cut off a vital route of energy supply. 2) This process can efficiently relieve the hypoxia condition, which probably will promote the glucose‐depletion by GOx. The catalytical capability of GOx can be evaluated by the producing‐level of H_2_O_2_. Therefore, to verify our hypothesis, the intracellular ROS levels in the cells that were incubated with CHC@ZIF‐8, GOx@ZIF‐8, and CHC/GOx@ZIF‐8 in the media supplied with glucose (10 mm) and lactate (10 mm) were analyzed by DCFH‐DA. According to the LSCM analysis (Figure 5C), compared with the control group, CHC@ZIF‐8 treatment caused a negligible increase in ROS while pronounced ROS generation was detected in the GOx@ZIF‐8 and CHC/GOx@ZIF‐8 treated groups. More importantly, the intracellular ROS level of cells treated with CHC/GOx@ZIF‐8 was much higher than that treated with GOx@ZIF‐8, which was further confirmed by FCM analysis (Figure 5D; Figure S15, Supporting Information). The exact reactive oxygen species was determined to be H_2_O_2_ by using the specific Hydrogen Peroxide Assay Kit (Figure S16, Supporting Information), and no obvious signals of singlet oxygen or hydroxyl radical were observed by using SOSG and APF as the fluorescence probes. Thus, we demonstrate that the introduction of CHC could improve the catalytical capability of GOx, consequently, promote glucose depletion and the generation of toxic H_2_O_2_, both of which would bring cell apoptosis. The finding reveals that it is a synergistically enhanced therapeutic strategy rather than a simple combination of two energy‐blocking approaches (Figure 4F).

### 2.6 Intracellular Therapeutic Efficacy of CHC/GOx@ZIF‐8

In this part, cells treated with different materials were stained with Annexin V‐FITC and PI to test the cellular apoptosis. According to the FCM analysis in Figure 5E, the group treated with CHC@ZIF‐8 showed negligible cell‐apoptosis, which was similar to the control group. It is probably because the energy can be compensated through the glucose‐influx route. 44.3% of cell‐apoptosis was observed for the group treated with GOx@ZIF‐8. Although the energy‐supply can be partially compensated from the exogenous lactate, the catalytical reaction of GOx produced cytotoxic H_2_O_2_. Finally, an apoptosis of up to 71.7% was observed for the group treated with CHC/GOx@ZIF‐8, which was attributed to the dual depriving of lactate and glucose. Cells were stained with Calcein‐AM/propidium iodide to label the live/dead cells to further characterize the improved therapeutic efficacy by CHC/GOx@ZIF‐8. According to the LSCM analysis in Figure 5F, few dead cells in the control group or CHC@ZIF‐8 group were detected. Approximately 50% of the cells treated with GOx@ZIF‐8 were dead, while more than 80% of the cells treated with CHC/GOx@ZIF‐8 were killed, which further confirmed the CHC/GOx@ZIF‐8 could induce improved cellular apoptosis and subsequent cell death. To summarize, the improved starvation therapy efficiency by CHC/GOx@ZIF‐8 should be reasoned to the dual functions of inhibiting the lactate influx, that is, 1) blocking the lactate‐fueled energy compensation when glucose was depleted by GOx; 2) improving the intra‐cellular O_2_ pressure to enhance the catalytic activity of GOx with more efficient glucose depletion and ROS generation.

### 2.7 In Vivo Therapeutic Efficacy of CHC/GOx@ZIF‐8

**Figure 6:**
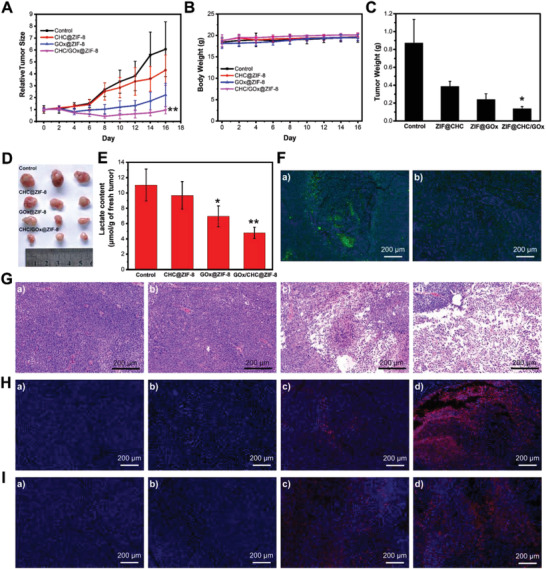
Efficient cancer starvation therapy by CHC/GOx@ZIF‐8 in vivo. A) Tumor growth curves and B) mice bodyweights after different treatment. C) Tumor weight and D) the corresponding photograph 16 days after different treatment. E) Lactate content in fresh tumors 24 h after different treatment. F) Immune fluorescence analysis of HIF‐1*α* of tumors 24 h after treatment with PBS and CHC/GOx@ZIF‐8. G) H&E staining, H) TUNEL analysis, and I) immune‐fluorescence analysis of caspase‐3 of tumors 24 h after the treatment with a) PBS, b) CHC@ZIF‐8, c) GOx@ZIF‐8, d) and CHC/GOx@ZIF‐8. Scale bar: 200 µm. Data are presented as mean ± SD, *n* = 3, *p* < 0.05, *p < 0.01.

Inspired by the effective starvation therapy by CHC/GOx@ZIF‐8 in vitro, we further conducted the in vivo tests. SiHa cervix xenograft tumor model, which also highly expresses MCT1 protein, was fabricated on BALB/c‐Nude mice. As a vital factor for in vivo applications, the biocompatibility of nanocrystals was tested by hemolysis assay (Figure S17, Supporting Information). There was no detectable hemolytic reaction even when the CHC/GOx@ZIF‐8 concentration was up to 100 µg mL^−1^. When the tumor size reached 200 mm^3^, CHC@ZIF‐8, GOx@ZIF‐8, CHC/GOx@ZIF‐8, and the equal volume of PBS were administrated by intratumor injection. The tumor size and bodyweight of mice were monitored for a period of 16 days. According to the tumor growth curve (**Figure** **6**A), inhibited tumor growth by CHC@ZIF‐8 was observed until ten days post‐administration, which is probably because the CHC inhibited lactate‐fueled ATP synthesis can be partially compensated by glucose at the early stage of drug treatment. For the GOx@ZIF‐8 treated group, the tumor growth was significantly inhibited, which should be a result of the GOx induced glucose starvation and toxic H_2_O_2_ generation. The CHC/GOx@ZIF‐8 treatment exhibited a more efficient inhibitory‐effect on tumor growth than GOx@ZIF‐8. According to Figure 6B, administration of nano drugs didn't influence the bodyweight of the mice comparing with the control group, which indicated no harmful side effect was caused by the drugs. At the end of tumor growth curve management, the mice were sacrificed, and the remaining tumors were analyzed. According to Figure 6C,D, both the tumor weight and size of the CHC/GOx@ZIF‐8 group were most significantly reduced than the other groups. Moreover, we measured the intra‐tumoral lactate level 24 h after the administration of different nanocrystals. As shown in Figure 6E, the intra‐tumoral lactate level after treatment with CHC@ZIF‐8 was lower than the control group. GOx@ZIF‐8 treatment significantly reduced the intra‐tumoral lactate level for the GOx mediated depletion of glucose, which is the precursor of lactate. For the tumors treated with CHC/GOx@ZIF‐8, a further decrease of the intra‐tumoral lactate level was observed, which revealed the influx of exogenous lactate was efficiently inhibited by CHC in vivo. Further analysis of the expression of HIF‐1*α* (hypoxia marker) in tumor tissues (Figure 6F) revealed the improved O_2_ pressure by the administration of CHC@ZIF‐8 into the tumor tissue, which confirmed blocking the lactate‐fueled respiration could relieve the hypoxia condition of solid tumors in vivo.

To further analyze the tumor growth inhibitory effect, histological analysis of the tumors was conducted. According to the H&E analysis, no cell death was observed in the tumor tissues treated with PBS and CHC@ZIF‐8 (Figure 6G). While more severe tumor cell damage was observed in the CHC/GOx@ZIF‐8 treated group, which is due to the synergistic effect of CHC mediated lactate starving and GOx catalyzed glucose starving. The improved cellular apoptosis by CHC/GOx@ZIF‐8 was also confirmed by terminal deoxynucleotidyl transferase‐mediated dUTP‐biotin nick end labeling (TUNEL) assay (Figure 6H). In addition to the starvation effect caused by glucose depletion and lactate blocking, H_2_O_2_ generated by GOx can also cause cell death by the pathway of ROS‐induced caspase‐3 activation, which is an executive protein for cellular apoptosis.^[^ ^26^ ^]^ Compared with other groups, the CHC/GOx@ZIF‐8 treatment induced the highest caspase‐3 expression (Figure 6I; Figure S18, Supporting Information), indicating that the greatest cell apoptosis was induced by the synergistic effect of CHC and GOx. Thus, the CHC/GOx@ZIF‐8 improved the starvation therapy efficiency in vivo by blocking the exogenous lactate influx and increasing the O_2_ pressure for improved GOx mediated glucose oxidation. As a result, the tumors were killed through the following two paths: 1) the starvation effect by depriving of both lactate and glucose and 2) cytotoxicity induced by elevated ROS levels. Finally, the main organs of mice treated with PBS and CHC/GOx@ZIF‐8 were subjected to H&E staining analysis (Figure S19, Supporting Information). Compared with PBS treated group, all the organs of mice treated with CHC/GOx@ZIF‐8 were normal, which indicated the good safety of CHC/GOx@ZIF‐8 for in vivo starvation applications.

## 3 Conclusions

In summary, for the purpose of enhancing the anti‐tumor efficacy of the cancer starvation therapy, a new nanomedicine, CHC/GOx@ZIF‐8, which is capable of dual‐depriving lactate and glucose, was designed and synthesized. The hydrophobic CHC and hydrophilic GOx were co‐loaded into ZIF‐8 by the one‐pot synthesis method. The introduction of DMF and increasing the usage of 2‐MIM were found vital for achieving the monodispersed nanomedicines. We experimentally proved that both lactate and glucose could fuel cell growth under the condition that the other one was under a shortage. Therefore, the simultaneous depriving of lactate and glucose could reinforce the effect of the starvation therapy. Moreover, we found that the intracellular ROS level of cells treated with CHC/GOx@ZIF‐8 was much higher than that treated with GOx@ZIF‐8, which also counted for the enhanced anti‐tumor efficacy. We revealed that CHC/GOx@ZIF‐8 could interfere with the lactate‐fueled respiration process and relieved the hypoxia condition tumors, which subsequently improved the catalytic activity of GOx, resulting in a more thorough glucose depletion and a higher ROS stress. Therefore, we disclosed that the synergistically enhanced anti‐tumor effect by CHC/GOx@ZIF‐8 was due to the dual starvation effect and the extra improved ROS level. Hemolysis assay and histological analysis of the organs of the mice treated with CHC/GOx@ZIF‐8 revealed the good biocompatibility of CHC/GOx@ZIF‐8. All these good results demonstrate that, under this advanced starvation therapeutic strategy, CHC/GOx@ZIF‐8 is a promising anti‐tumor nanomedicine showing great potentials in future clinical applications.

## 4 Experimental Section

### Synthesis of ZIF‐8

1.135 g of 2‐MIM was first dissolved by 4.0 g of water, then 58.5 mg of Zn(NO_3_)_2_∙6H_2_O dissolved by 0.4 g of water was quickly injected into the 2‐MIM solution under vigorous stirring at room temperature. Ten minutes later, the formed ZIF‐8 crystals were separated from the reagents by centrifugation at a speed of 13 200 rpm for 10 min, washed with water twice, and re‐dispersed in water.

### Synthesis of GOx@ZIF‐8

1.135 g of 2‐MIM was first dissolved by 4.0 g of water, then 6 mg of GOx was added into the 2‐MIM solution. After stirring for 5 min, 58.5 mg of Zn(NO_3_)_2_∙6H_2_O dissolved by 0.4 g of water was quickly injected into the 2‐MIM solution under vigorous stirring at room temperature. After another stirring for 10 min, the formed crystals were separated by centrifugation (13 200 rpm, 10 min), washed twice with water, and finally redispersed in water.

### Lactate‐Fueled Cellular Survival Test

Cells were seeded at a density of 10 000 cells per well and incubated 24 h before the tests. To characterize the lactate mediated cell survival without other nutrition supply and incubated with different concentrations of lactate (0, 1, 2.5, 5, 10, 15, and 20 mm) in DMEM cell culture media. Cell proliferation and lactate concentrations in the supernatants were measured by CCK‐8, LA assay kit according to the manufacturer's instructions respectively. To test the lactate‐mediated cellular survival while glucose was depleted by GOx@ZIF‐8, different concentrations of GOx@ZIF‐8 were dispersed in the DMEM media supplied with glucose (10 mm) or media supplied with glucose (10 mm) and lactate (5 mm) and incubated with the cells for different periods. Then, cell proliferation was measured by CCK‐8 assay.

### Synthesis of CHC@ZIF‐8 Using DMF as a Co‐Solvent

1.784 g of 2‐MIM was first dissolved by 4.0 g of water, then 16 mg of CHC and 720.8 mg of DMF were added. After stirring for 5 min, 58.5 mg of Zn(NO_3_)_2_∙6H_2_O dissolved by 0.4 g of water was quickly injected into the above solution under vigorous stirring at room temperature. After another stirring for 10 min, the formed crystals were separated by centrifugation (13 200 rpm, 10 min), washed with ethanol twice, washed with water twice, and finally redispersed in water.

### Synthesis of CHC/GOx@ZIF‐8 Using DMF as a Co‐Solvent

1.784 g of 2‐MIM was first dissolved by 4.0 g of water, then 16 mg of CHC, 720.8 mg of DMF, and 6 mg of GOx were added. After stirring for 5 min, 58.5 mg of Zn(NO_3_)_2_∙6H_2_O dissolved by 0.4 g of water was quickly injected into the above solution under vigorous stirring at room temperature. After another stirring for 10 min, the formed crystals were separated by centrifugation (13 200 rpm, 10 min), washed with 10% ethanol v/v twice, washed with water twice, and finally redispersed in water.

### Loading Capacity of CHC and GOx in ZIF‐8

A comparison method was used to calculate the loading capacity of CHC in CHC/ZIF‐8 and CHC/GOx@ZIF‐8. During the synthesis and purification process, all the supernatants after removing the precipitated products were collected together. Then the absorption spectra of the supernatant mixes were measured to record the absorbance of CHC. The group that was not possible to form ZIF‐8 by the removal of Zn^2+^ was set as the control and was treated the same as the sample group. Weights of the purified products were determined after freeze‐drying. Finally, the loading capacity was calculated according to Equation (1)

(1) $$\mathrm{wt}\%\;=\frac{A_{c}-A_{s}}{A_{c}}\;\times \frac{16\mathrm{mg}}{W}\times \;100\%$$

where *A*_s_ represents the absorbance of CHC in supernatants of the “sample group”, *A*_c_ represents the absorbance of CHC in supernatants of the “control group”, and *W* represents the weight of the purified product.

The loading capacity of GOx in ZIF‐8 was calculated according to the SDS‐PAGE analysis. The final volume of the synthesized products was adjusted to 4 mL, and 10 µL of the final product was subjected to SDS‐PAGE analysis. The intensity of the GOx band was quantified by using the Image J 2X software, and the amount of GOx was calculated according to the calibration curve of GOx based on SDS‐PAGE. The loading capacity of GOx in GOx@ZIF‐8 or CHC/GOx@ZIF‐8 was calculated according to Equation (2)

(2) $$\mathrm{wt}\%\;=\frac{W_{s}}{W}\;\times \;400\;\times \;100\%$$

where *W*_s_ represents the weight of GOx determined by the SDS‐PAGE analysis, and *W* represents the weight of the final product.

### SDS‐PAGE Analysis

The synthesized GOx@ZIF‐8 and CHC/GOx@ZIF‐8 were disassembled by EDTA and heated at 95 °C for 5 min. The proteins were subjected to electrophoretic analysis with an 8% SDS‐PAGE gel. The gel was then stained by Coomassie brilliant blue G 250 for 4 h and rinsed by the distaining solution (volume ratio of methanol:glacial acetic acid:water = 5:1:4).

### Evaluation of the Weak Acidic Environment Triggered GOx Release and the Catalytic Activity after Release

AF‐647 labeled GOx was used to synthesis CHC/GOx@ZIF‐8. To test the GOx release under acidic conditions, 50 µg mL^−1^ of CHC/GOx@ZIF‐8 was dispersed in PBS with different pH values (5.2, 6.0, and 7.4) and incubated at 37 °C. The supernatants were collected by centrifugation every one hour by centrifugation. The fluorescence intensities of AF‐647 were measured to monitor the released GOx. To test the catalytic activity of GOx in CHC/GOx@ZIF‐8, the synthesized CHC/GOx@ZIF‐8 was dispersed in PBS (pH = 5.2) and incubated at 37 °C for 6 h. The amount of GOx was quantified by measuring the fluorescence of AF 647. Then GOx (0.2 µg mL^−1^), HRP (0.1 µg mL^−1^), TMB (200 nm), and glucose (2 mm) were mixed in PBS (pH = 5.2) and incubated at 37 °C. The generation of OxTMB was monitored by measuring the absorbance at 649 nm.

### CHC@ZIF‐8 Mediated Blocking of the Lactate‐Fueled Respiration Test

Cells were seeded at a density of 10 000 cells per well in 96‐well plates and incubated 24 h before the tests. Different concentrations (0, 10, 20, and 30 µg mL^−1^) of CHC@ZIF‐8 and corresponding CHC were incubated with cells for different periods in MEM media containing 10 mm of lactate. Then, cell proliferation and lactate concentrations in the supernatants were measured by the CCK‐8 assay and LA assay, respectively. To test the oxygen consumption rate, cells were first incubated with 20 µg mL^−1^ of CHC@ZIF‐8 and the corresponding amount of CHC for 12 h. Then the cells were subjected to the OCR assay according to the manufacturer's instructions.

### Starvation Therapy Test at the Co‐Existence of Lactate and Glucose

Cells were seeded at a density of 10 000 cells per well in 96‐well plates and incubated 24 h before the tests. Different concentrations (0, 10, 20, 30, and 40 µg mL^−1^) of ZIF‐8, CHC@ZIF‐8, GOx@ZIF‐8, and CHC/GOx@ZIF‐8 were incubated with cells in the DMEM cell culture medium with 10 mm of glucose and 10 mm of lactate. Then, cell proliferation was measured every 24 h by CCK‐8 assay for the period of 96 h.

### ROS Detection In Vitro

Cells were seeded at a density of 10 000 cells per well in a 96‐well plate for LSCM analysis and 200 000 cells per well in a 6‐well plate for FCM analysis. Cells were incubated at 37 °C for 24 h to allow complete adherence. Then 20 µg mL^−1^ of CHC@ZIF‐8, GOx@ZIF‐8, and CHC/GOx@ZIF‐8 were incubated with cells in DMEM media supplied with glucose (10 mm) and lactate (10 mm) for 12 h. Then cells were washed with PBS and stained with DCFH‐DA (1 µm) in PBS for 30 min. For LSCM analysis, the fluorescence of DCF was excited by 488 nm laser, and emission was collected from 510 to 550 nm. For FCM analysis, cells were dissociated by trypsin and dispersed in PBS. The fluorescence of DCF was collected from 10 000 cells and analyzed by the FloJo‐V10.

### Cellular Apoptosis Analysis

Cells were seeded at density of 200 000 cells per well in 6‐well plate and incubated for 24 h. Then 20 µg mL^−1^ of CHC@ZIF‐8, GOx@ZIF‐8, and CHC/GOx@ZIF‐8 were incubated with cells in DMEM media supplied with glucose (10 mm) and lactate (10 mm) for 12 h. Then cells were dissociated and co‐stained by Annexin V‐FITC and PI according to the manufacturer's instructions. Then fluorescence of FITC and PI were collected from 10 000 cells and analyzed.

### Double Fluorescence Mark Assay for Detection of Cell Death

Cells were seeded at a density of 10 000 cells per well in a 96‐well plate and incubated for 24 h. Then 20 µg mL^−1^ of CHC@ZIF‐8, GOx@ZIF‐8, and CHC/GOx@ZIF‐8 were incubated with cells in DMEM media supplied with glucose (10 mm) and lactate (10 mm) for 12 h. Then cells were stained with CAM and PI according to the manufacturer's instructions. CAM and PI were excited by the 488 and 552 nm laser, respectively. Emission of CAM and PI were collected at 500—540 nm and 615—645 nm.

### In Vivo Starvation Therapy

All the animal experiments were conducted according to the Institutional Animal Use and Care Regulations (protocol no. SUSTC‐JY2019068) approved by the Laboratory Animal Ethics Committee of SUSTech. All the animals were acclimated and tested for infectious diseases for one week since they were received. To fabricate the subcutaneous tumor, 3 × 10^6^ cells were injected into the posterior flank regions of the mice. The tumors were measured by a vernier caliper and calculated according to the formula: Volume = (Length × Width × Width)/2.

When the tumor size reached 200 mm^3^, the mice were randomly divided into 4 groups. PBS, CHC@ZIF‐8, GOx@ZIF‐8, and CHC/GOx@ZIF‐8 were intratumorally injected into the tumors. Histological analysis of the tumor tissues was conducted 24 h after the injection of drugs. The tumor size and bodyweight of the mice were recorded every two days after the injection. 16 days after the drug administration, the mice were sacrificed, and major organs (lung, heart, liver, kidney, and spleen) were used for the tissue slides and histopathological assessment. The isolated tumors were weighted and imaged.

To measure the intra tumoral lactate level, tumors were isolated and ground 24 h after the drug administration. Then, the lactate concentration was measured by using the LA assay kit. Histopathological assessment, TUNEL analysis, and immunofluorescence analysis were conducted according to previous studies.^[^ ^27^ ^]^

### Statistical Analysis

The cell proliferation (%) was calculated by normalizing average OD450 of the control group to 100%. All the values are presented as mean ± SD, and the data were obtained based on three independent tests. *p*‐values were calculated according to the analysis of variance (ANOVA) with Bonferroni correction by using the SPSS 19.0 software, *p < 0.05, **p < 0.01, ***p < 0.001. Diameters of nanoparticles were calculated using the Nano Measure 2.0 software. Fluorescence intensity analysis of the images was conducted using the Image J 2X software. The data of FCM was analyzed by FloJo‐V10.

## Conflict of Interest

The authors declare no conflict of interest.

## Supporting information

Supporting Information

## Data Availability

The data that support the findings of this study are available from the corresponding author upon reasonable request.
