# Supplementary material for: Advanced Cancer Starvation Therapy by Simultaneous Deprivation of Lactate and Glucose Using a MOF Nanoplatform
Source: Adv Sci (Weinh). 2021 Aug 7;8(19):2101467. doi: 10.1002/advs.202101467 (PMC8498878; doi:10.1002/advs.202101467)
Supplement: Supplementary file 1 — Supporting Information [file ADVS-8-2101467-s001.pdf]

© 2021 Wiley-VCH GmbH

ADVANCED SCIENCE
Open Access

Supporting Information

for *Adv. Sci.*, DOI: 10.1002/advs.202101467

Advanced Cancer Starvation Therapy by Simultaneous Deprivation of Lactate and Glucose Using a MOF Nanoplatform

Jiantao Yu, Zixiang Wei, Qing Li, Feiyan Wan, Zhicong Chao, Xindan Zhang, Li Lin, Hong Meng and Leilei Tian**

Supporting Information

Advanced Cancer Starvation Therapy by Simultaneous Deprivation of Lactate and Glucose Using a MOF Nanoplatform

Jiantao Yu, Zixiang Wei, Qing Li, Feiyan Wan, Zhicong Chao, Xindan Zhang, Li Lin, Hong Meng and Leilei Tian**

Contains

Supporting methods……………………………………...……………………….....….... S3

Figure S1. (A) TEM image and (B) corresponding size distribution of ZIF-8 crystals synthesized at Zn^{2+}: 2-MIM: H_2O ratio of 1: 70: 1245……………………………………...S10

Figure S2. SEM images of CHC@ZIF-8 crystals synthesized at Zn^{2+}: CHC: H_2O molar ratio of 1: 0.36: 1245 with varying amount of 2-MIM…………………………………………… S11

Figure S3. SEM images of CHC@ZIF-8 crystals synthesized at Zn^{2+}: CHC: 2-MIM: H_2O molar ratio of 1: 0.36: 110: 1245 with varying amount of DMF……………………….…S12

Figure S4. (A) TEM image and (B) corresponding size distribution of CHC@ZIF-8 crystals synthesized at Zn^{2+}: 2-MIM: CHC: H_2O: DMF ratio of 1: 110: 0.36: 1245: 50.……………...S13

Figure S5. SEM images of CHC@ZIF-8 crystals synthesized at Zn^{2+}: CHC: 2-MIM: H_2O molar ratio of 1: 0.36: 110: 1245 with varying amount of (A) TEA, (B) methanol, (C) ethanol and (D) DMSO.………………………………………………………...…………………… S14

Figure S6. Cell proliferation after incubation with CHC/GOx@ZIF-8 containing different amount of natural GOx…….………….…………………………......….……………….…S15

Figure S7. Absorbance spectra of CHC in the supernatants during the synthesis of CHC@ZIF-8 and CHC/GOx@ZIF-8..S16

Figure S8. (A) LSCM and (B) FCM analysis of the cellular internalization of CHC/GOx@ZIF-8 (20 μg/mL). (C) Corresponding mean fluorescence intensity according to FCM analysis…S17

Figure S9. Cell proliferation after incubation with CHC in medium supplied with lactate (10 mM) for (A) 24, (B) 48, (C) 72 and (D) 96 hours…………………………………………S18

Figure S10. Cell proliferation after incubation with CHC in the medium supplied with glucose (10 mM) for different periods……………………………..S19

Figure S11. Cell proliferation after incubation with CHC@ZIF-8 in the medium supplied with lactate (10 mM) for different periods…………………………………………..………S20

Figure S12. Cell proliferation after incubation with different concentrations of ZIF-8 for different periods in DMEM supplied with glucose (10 mM) and lactate (10 mM).……....…S21

Figure S13. Cell proliferation after incubation with different concentrations of ZIF-8 for different periods in DMEM supplied with 10% FBS and using (A) glucose (10 mM) and (B) lactate (10 mM) as a single carbon source…..…S22

Figure S14. LSCM images of cells stained with Hypoxia Red after different treatment ………………………………………………………………………………………S23

Figure S15. Mean fluorescence intensity of DCF according to the FCM analysis of cells after different treatments…………………...……………………………………………………S24

Figure S16. LSCM images of cells stained with (A) AbGreen, (B) APF and (C) SOSG after different treatment.……………………………………………………………………... S25

Figure S17. Hemolytic activities of CHC/GOx@ZIF-8 (2–100 μg/mL) suspensions toward RBCs in PBS...S26

Figure S18. Quantitative fluorescence intensity of antibodies labeling the caspase-3 in Figure 6I using the Image J 2X software...S27

Figure S19. H & E staining of major organs of the mice 16 days after treatment with PBS and CHC/GOx@ZIF-8. All the images were achieved under the same magnification…………...S28

Supporting methods

Chemicals: L-Sodium lactate, L-lactate acid, 2-methylimidazole (2-MIM), N, N-Dimethylformamide (DMF), triethylamine (TEA), dimethyl sulfoxide (DMSO), methanol, ethanol, heparin sodium, and ethylenediaminetetraacetic acid disodium salt dihydrate (EDTA) were purchased from Aladdin Chemistry (Shanghai, China). $Zn(NO_3)_2{\cdot}6H_2O$ was purchased from Macklin (Shanghai, China). α-cyano-4-hydroxycinnamate (CHC) and DCFH-DA were purchased from Sigma-Aldrich (Shanghai, China). Tris (hydroxymethyl) aminomethane (Tris), sodium dodecyl sulfate (SDS), D-glucose, peroxidase (HRP), and glucose oxidase (GOx) were purchased from Sangon Biotech (Shanghai, China). Singlet oxygen sensor green (SOSG), Fetal bovine serum (FBS), trypsin, penicillin/streptomycin, protein ladder (10 - 180 kDa), Alexa Fluor™ 647 (AF 647) NHS ester and Dulbecco's Modified Eagle's Medium (DMEM) without glucose were purchased from Thermo Fisher Scientific (Beijing, China). Hydrogn peroxide detection kit was purchased from Abcom (Shanghai, China). ROS-ID® Hypoxia/Oxidative Stress Detection Kit was purchased from Enzo Life Sciences (New York, USA). Oxygen consumption rate (OCR) kit was purchased from Cayman Chemicals (Michigan, USA). 30% acrylamide-bisacrylamide stock solution, Annexin V-FITC apoptosis detection kit, coomassie brilliant blue G250, and Hoechst 33342 were purchased from Beyotime Biotechnology (Shanghai, China). 3,3′,5,5′-Tetramethylbenzidine (TMB) was purchased from MedChemExpress (Shanghai, China). LA assay kit, cell counting kit (CCK-8), glycine, and Calcein-AM/PI cell live/dead staining kit were purchased from Solarbio Life Science (Beijing, China). Aminophenyl fluorescein (APF) was purchased from Shanghai Maokang Biotechnology (Shanghai, China). MCF-7 (human breast cancer) cells were donated by Professor Ying Sun at the Department of Biology of SUSTech. Terminal deoxynucleotidyl transferase-mediated dUTP-biotin nick end labeling (TUNEL) assay kit, antibody of HIF-1α, and antibody of caspase-3 were purchased from Servicebio Life Science (Wuhan, China). Siha (human cervical carcinoma) cells were purchased from ATCC (American type culture

collection). Mice were purchased from Beijing Vital River Laboratory Animal Technology Co., Ltd. (Beijing, China).

Apparatus: TEM images of the synthesized crystals were obtained from a Hitachi HT7700 transmittance electron microscope (Hitachi, Japan) operated at 100 KV. SEM images of the synthesized crystals were obtained from a Mira3 field emission scanning electron microscope operated at 10 KV (Tescan, Czechia). Hydrodynamic size and zeta potential measurements were conducted on a Nano ZS Zetasizer (Malvern Instruments, UK). FTIR spectra were collected on a Frontier Spectrometer (PerkinElmer, USA). PXRD analysis was conducted on a D8 Focus diffractometer (Rigaku SmartLab, Japan) using Cu-Kα radiation. TGA analysis was conducted on a Discovery TGA 5500 (TA Instrument, USA). N_2 sorption isotherms were recorded by using an ASAP-2046 surface area/pore size analyzer (Micromeritics, USA). UV-vis spectra were collected by using a UV-2600 spectrophotometer (Shimadzu Instruments, Japan). SDS-PAGE analysis was conducted on a Bio-Rad miniprotern®Tetra system (Bio-Rad, USA). Fluorescent images of cells were taken on a TCS SP8 LCSM (Leica, Germany). FCM was conducted by using a FACSCanto Analyzer (BD Instrument, USA). Cytation 3 plate reader (BioTek, USA) The absorbance of CCK-8 assay, LA assay, TMB oxidation assay, the fluorescence of pH triggered AF-647 labeled protein release, and the phosphorescence of the OCR assay, all were measured by a Cytation 3 plate reader (BioTek, USA). All the photographs were taken by a Nikon D3500 camera (Nikon, Japan). The TUNEL, H&E, immunofluorescence slides were imaged by the Leica Aperio Digital Pathology imaging system (Leica, Germany).

Direct loading of CHC by ZIF-8: 1.135 g of 2-MIM was firstly dissolved by 4.0 g of water, then 12 mg, 16 mg, and 20 mg of CHC were added into the 2-MIM solution, respectively. After stirring for 5 min, 58.5 mg of $Zn(NO_3)_2{\cdot}6H_2O$ dissolved by 0.4 g of water was quickly injected into the 2-MIM solution under vigorous stirring at room temperature. After another stirring for 10 minutes, the formed crystals were separated by centrifugation (13,200 rpm, 10 min), washed with ethanol twice, washed with water twice, and finally redispersed in water.

Increase the usage of 2-MIM to adjust the size of CHC@ZIF-8: The molar ratio of Zn^{2+}: CHC: water was fixed to 1: 0.36: 1245, while the 2-MIM: Zn^{2+} ratios were 90: 1 and 110: 1, respectively. 1.459 g or 1.784 g of 2-MIM were firstly dissolved by 4.0 g of water, then 16 mg of CHC was added into the 2-MIM solution. After stirring for 5 min, 58.5 mg of $Zn(NO_3)_2{\cdot}6H_2O$ dissolved by 0.4 g of water was quickly injected into the 2-MIM solution under vigorous stirring at room temperature. After another stirring for 10 minutes, the formed crystals were separated by centrifugation (13,200 rpm, 10 min), washed with ethanol twice, and characterized by SEM.

Use DMF to adjust the size of CHC@ZIF-8: The molar ratio of Zn^{2+}: 2-MIM: CHC: water was fixed to 1: 70: 0.36: 1245. 1.135 g of 2-MIM was firstly dissolved by 4.0 g of water. Then 16 mg of CHC was added into the 2-MIM solution. 360.4 mg, 720.8 mg, and 1081.2 mg of DMF (The molar ratios of DMF: Zn^{2+} are 25: 1, 50:1, and 75:1, respectively) were added into the solution. After stirring for 5 min, 58.5 mg of $Zn(NO_3)_2{\cdot}6H_2O$ dissolved by 0.4 g of water was quickly injected into the 2-MIM solution under vigorous stirring at room temperature. After another stirring for 10 minutes, the formed crystals were separated by centrifugation (13,200 rpm, 10 min), washed with ethanol twice, and characterized by SEM.

Use TEA to adjust the size of CHC@ZIF-8: The molar ratio of Zn^{2+}: 2-MIM: CHC: water was fixed to 1: 110: 0.36: 1245. 1.784 g of 2-MIM was firstly dissolved by 4.0 g of water. Then 16 mg of CHC was added into the 2-MIM solution. 8.59 mg, 17.19 mg and 25.78 mg of TEA (the molar ratios of TEA: Zn^{2+} are 0.43: 1, 0.86:1, and 1.29:1, respectively) were added into the solution. After stirring for 5 min, 58.5 mg of $Zn(NO_3)_2{\cdot}6H_2O$ dissolved by 0.4 g of water was quickly injected into the 2-MIM solution under vigorous stirring at room temperature. After another stirring for 10 minutes, the formed crystals were separated by centrifugation (13,200 rpm, 10 min), washed with ethanol twice, and characterized by SEM.

Use methanol to adjust the size of CHC@ZIF-8: The molar ratio of Zn^{2+}: 2-MIM: CHC: water was fixed to 1: 110: 0.36: 1245. 1.784 g of 2-MIM was firstly dissolved by 4.0 g of water. Then 16 mg of CHC was added into the 2-MIM solution. 316 mg, 632 mg, and 948 mg of methanol

(the molar ratios of methanol: Zn^{2+} are 50: 1, 100:1, and 150:1, respectively) were added into the solution. After stirring for 5 min, 58.5 mg of $Zn(NO_3)_2{\cdot}6H_2O$ dissolved by 0.4 g of water was quickly injected into the 2-MIM solution under vigorous stirring at room temperature. After another stirring for 10 minutes, the formed crystals were separated by centrifugation (13,200 rpm, 10 min), washed with ethanol twice, and characterized by SEM.

Use ethanol to adjust the size of CHC@ZIF-8: The molar ratio of Zn^{2+}: 2-MIM: CHC: water was fixed to 1: 110: 0.36: 1245. 1.784 g of 2-MIM was firstly dissolved by 4.0 g of water. Then 16 mg of CHC was added into the 2-MIM solution. 636.9 mg, 955.4 mg, and 1273.8 mg of ethanol (the molar ratios of ethanol: Zn^{2+} are 70: 1, 105:1, and 140:1, respectively) were added into the solution. After stirring for 5 min, 58.5 mg of $Zn(NO_3)_2{\cdot}6H_2O$ dissolved by 0.4 g of water was quickly injected into the 2-MIM solution under vigorous stirring at room temperature. After another stirring for 10 minutes, the formed crystals were separated by centrifugation (13,200 rpm, 10 min), washed with ethanol twice, and characterized by SEM.

Use DMSO to adjust the size of CHC@ZIF-8: The molar ratio of Zn^{2+}: 2-MIM: CHC: water was fixed to 1: 110: 0.36: 1245. 1.784 g of 2-MIM was firstly dissolved by 4.0 g of water. Then 16 mg of CHC was added into the 2-MIM solution. 434.2 mg, 868.4 mg, and 1302.6 mg of DMSO(the molar ratios of DMSO: Zn^{2+} are 28.5: 1, 57:1, and 85.5:1, respectively) were added into the solution. After stirring for 5 min, 58.5 mg of $Zn(NO_3)_2{\cdot}6H_2O$ dissolved by 0.4 g of water was quickly injected into the 2-MIM solution under vigorous stirring at room temperature. After stirring for another 10 minutes, the formed crystals were separated by centrifugation (13,200 rpm, 10 min), washed with ethanol twice, and characterized by SEM.

Cell culture: MCF-7 and Siha cells were maintained with DMEM cell culturing medium supplied with 10% (v/v) of FBS, streptomycin (100 µg/mL), and penicillin (100 units/mL) at 37 °C. The air of the incubator was a humidified mixture of 95% ambient air and 5% CO_2. The cell culturing media was changed every 2 days, and the cells were subjected to a passage with trypsin every 4 days.

Cellular internalization of CHC/GOx@ZIF-8: Cells were seeded at a density of 10,000 cells per well in 96-well plates and incubated for 24 hours. AF 647 labeled GOx was used to synthesis the CHC/GOx@ZIF-8. 20 μg/mL of CHC/GOx@ZIF-8 were incubated with cells for different periods in DMEM cell culture media supplied with glucose (10 mM) and lactate (10 mM). For LSCM analysis, the cell nucleus was labeled by Hoechst 33342. AF-647 was excited by a 638-nm laser, and fluoresce emission was collected from 650 nm to 700 nm. For FCM analysis, the cells were dissociated by trypsin and washed with PBS twice, and finally resuspended in PBS. Fluorescence of AF 647 from 10,000 cells was collected and analyzed by the FlowJo-V10 software.

Cytotoxicity analysis of ZIF-8: when cells were cultured in media supplied with a single carbon source: Cells were seeded at a density of 10,000 cells per well in 96-well plates and incubated 24 hours before the tests. Different concentrations (0, 10, 20, 30, 40 μg/mL) of ZIF-8 were incubated with cells in the DMEM cell culture media supplied with 10 mM of glucose or 10 mM of lactate as a single carbon source. Then, cell proliferation was measured every 24 hours by CCK-8 assay for the period of 96 hours.

ROS species confirmation in vitro: Cells were seeded at a density of 10,000 cells per well in a 96-well plate and incubated at 37 °C for 24 hours to allow complete adherence. Then 20 μg/mL of CHC@ZIF-8, GOx@ZIF-8, and CHC/GOx@ZIF-8 were incubated with cells in DMEM media supplied with glucose (10 mM) and lactate (10 mM) for 12 hours. Then cells were washed with PBS and stained with APF (10 μM, in PBS), AbGreen (1X, in working solution) and SOSG (5 μM, in PBS) for 30 min. Then cells were washed with PBS and subjected to the LSCM analysis. For LSCM analysis, APF, AbGreen and SOSG were excited by 488 nm laser, and emission was collected from 510 nm to 550 nm.

Hypoxia condition analysis: Cells were seeded at a density of 10,000 cells per well in a 96-well plate and incubated at 37 °C for 24 hours to allow complete adherence. Then 20 μg/mL of CHC@ZIF-8 and corresponding amount of CHC were added to incubate with the cells for 12

hours. Then cells were stained with the Hypoxia-red probe of the ROS-ID® Hypoxia/Oxidative Stress Detection Kit according to the manufacturer's instructions. Then cells were subjected to LSCM analysis. For LSCM analysis, Hypoxia-red was excited by 552 nm laser, and emission was collected from 600 nm to 650 nm.

Synthesis of CHC/GOx@ZIF-8 with different ratios of CHC and natural GOx: GOx heated at 95 °C for 10 min was mixed with different amounts of natural GOx to make the GOx mixture of natural GOx and denatured GOx. 1.784 g of 2-MIM was firstly dissolved by 4.0 g of water, then 16 mg of CHC, 720.8 mg of DMF, and 8 mg of GOx mixture of natural GOx and denatured GOx were added. After stirring for 5 min, 58.5 mg of $Zn(NO_3)_2 \cdot 6H_2O$ dissolved by 0.4 g of water was quickly injected into the above solution under vigorous stirring at room temperature. After another stirring for 10 minutes, the formed crystals were separated by centrifugation (13,200 rpm, 10 min), washed with 10% ethanol (v/v) twice, washed with water twice, and finally redispersed in water.

Cell killing efficiency using CHC/GOx@ZIF-8 containing different ratios of CHC and active GOx: Cells were seeded at a density of 10,000 cells per well in 96-well plates and incubated 24 hours before the tests. 20 μg/mL of the synthesized CHC/GOx@ZIF-8 nano cryatals with different CHC and GOx ratios were incubated with cells in the DMEM cell culture media supplied with 10 mM of glucose and 10 mM of lactate for 24 hours. Then, cell proliferation was measured by CCK-8 assay.

Hemolysis assay: Fresh blood samples from a mouse were drawn from the eye into a tube containing EDTA. The red blood cells (RBCs) were separated from the serum by centrifugation (1000 rcf, 10 min, 4 °C). Then the RBCs were washed with PBS twice and stored at 4°C. Different concentrations of CHC/GOx@ZIF-8 were incubated with 4% RBCs for 2 hours at 37 °C. The RBCs incubated with PBS and water were set as negative and positive controls, respectively. Then the RBCs in tubes were centrifuged, and absorbance at 576 nm of the supernatants was measured. An equal volume of PBS was set as a blank for the absorbance

measurement. The hemolysis activity was calculated according to the formula: Hemolysis (%) = $(Abs_{sample} - Abs_{blank}) / (Abs_{water} - Abs_{blank}) \times 100\%$.

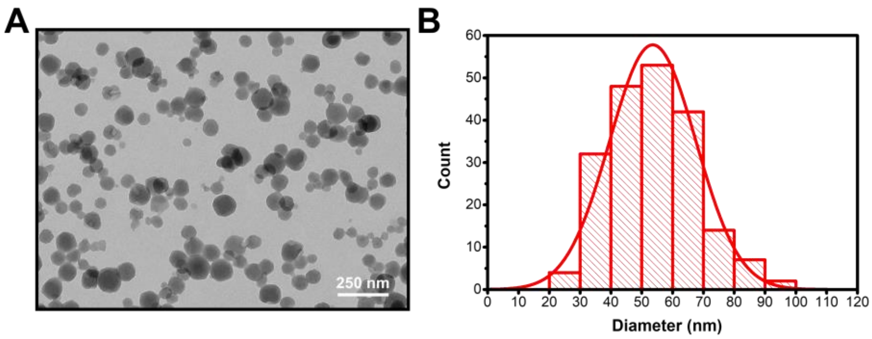

Figure S1. (A) TEM image and (B) corresponding size distribution of ZIF-8 crystals synthesized at Zn^{2+}: 2-MIM: H_2O ratio of 1: 70: 1245. Scale bar: 250 nm.

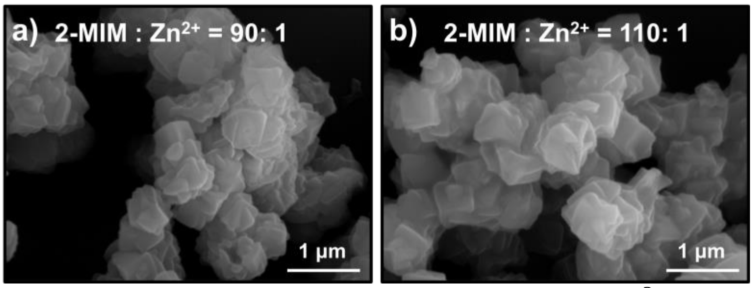

Figure S2. SEM images of CHC@ZIF-8 crystals synthesized at Zn^{2+}: CHC: H_2O molar ratio of 1: 0.36: 1245 with varying amount of 2-MIM. Scale bar: 1 μm.

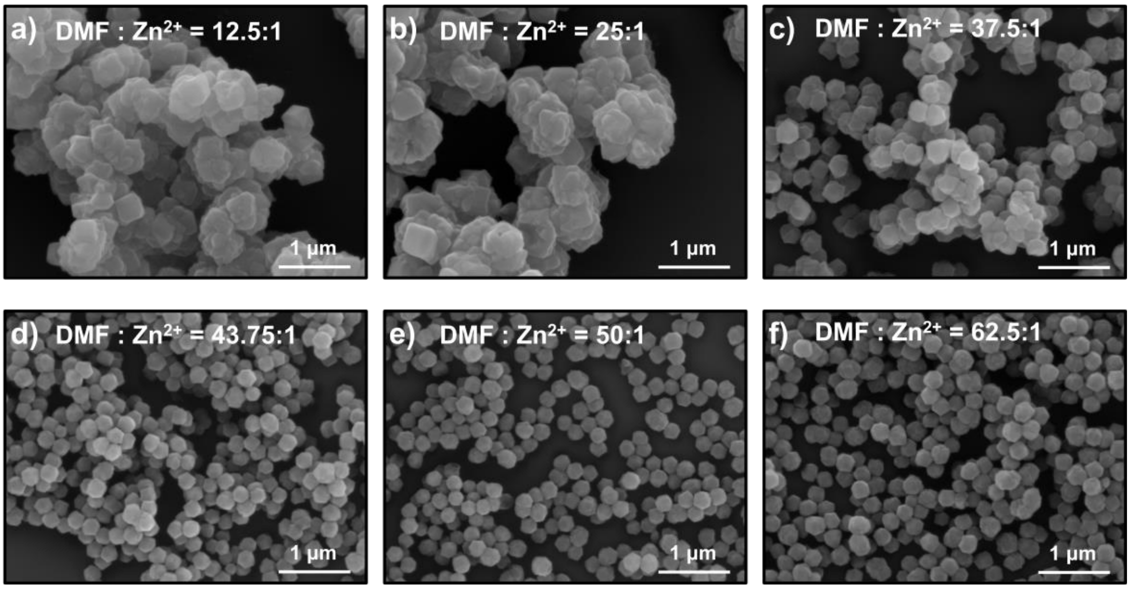

Figure S3. SEM images of CHC@ZIF-8 crystals synthesized at Zn^{2+}: CHC: 2-MIM: H_2O molar ratio of 1: 0.36: 110: 1245 with varying amount of DMF. Scale bar: 1 µm.

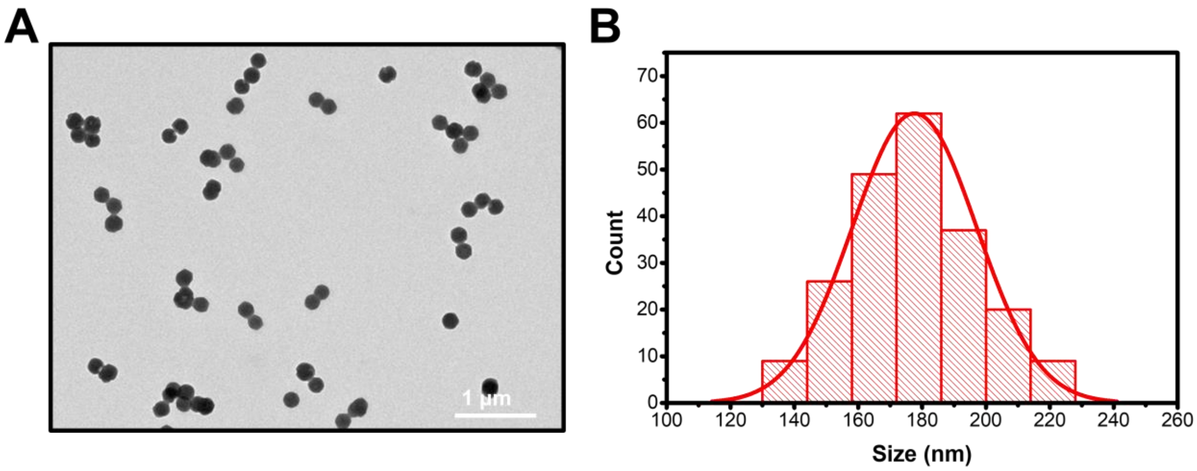

Figure S4. (A) TEM image and (B) corresponding size distribution of CHC@ZIF-8 crystals synthesized at Zn^{2+}: 2-MIM: CHC: H_2O: DMF ratio of 1: 110: 0.36: 1245: 50. Scale bar: 1 µm.

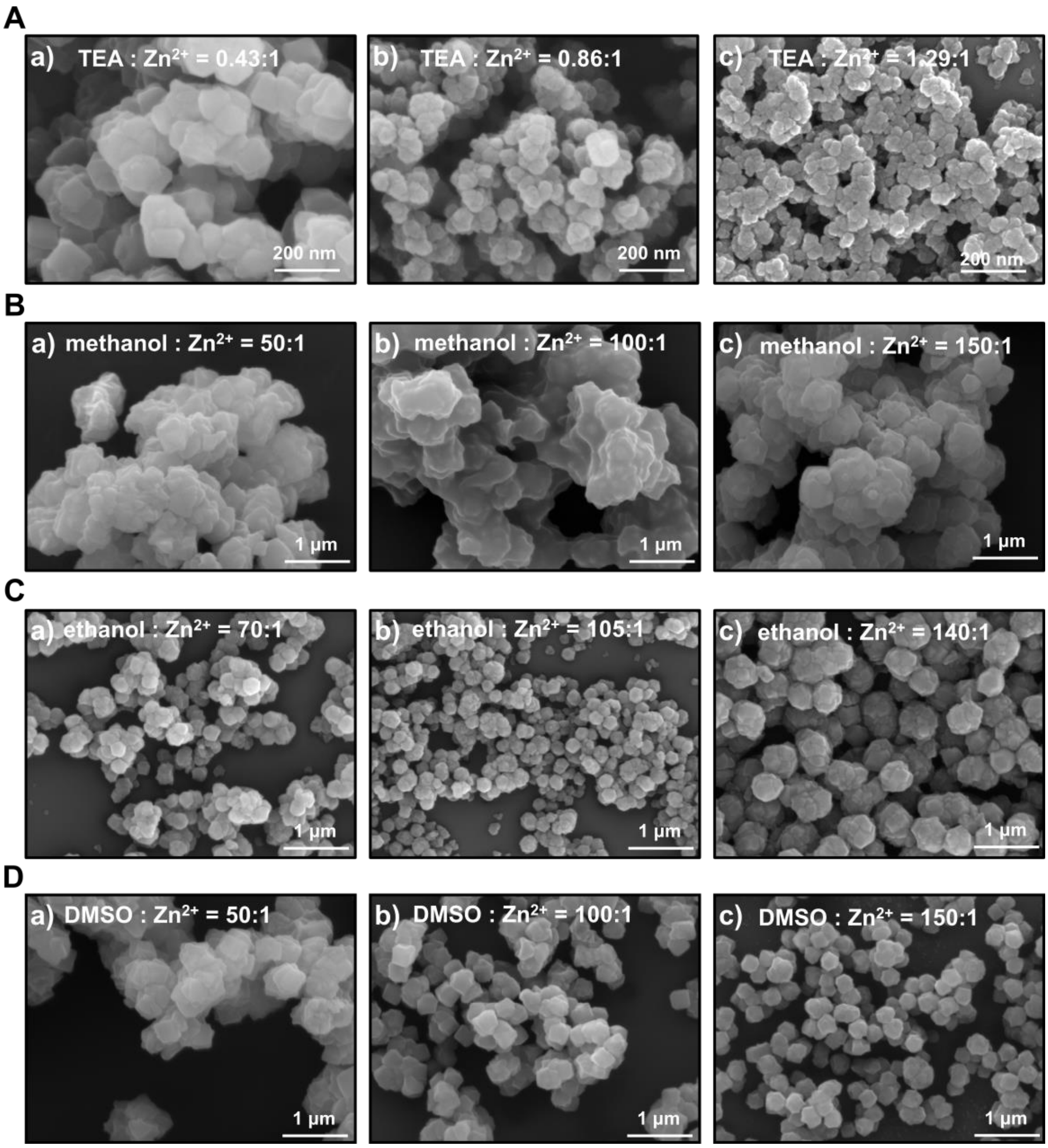

Figure S5. SEM images of CHC@ZIF-8 crystals synthesized at Zn^{2+}: CHC: 2-MIM: H_2O molar ratio of 1: 0.36: 110: 1245 with varying amount of (A) TEA, (B) methanol, (C) ethanol and (D) DMSO. Scale bar: 1 μm.

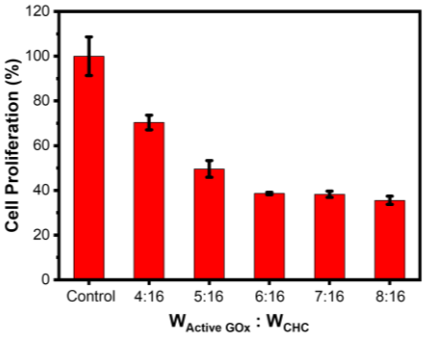

Figure S6. Cell proliferation after incubation with CHC/GOx@ZIF-8 containing different amount of natural GOx.

The influence of the ratios between CHC and GOx on their synergistic effect was investigated. However, both the amounts of CHC and GOx will significantly alter the morphology of the resultant CHC/GOx@ZIF-8 and its biomedical properties, which put some difficulties toward the experimental design. Therefore, considering the greater impact of CHC on the morphology of CHC/GOx@ZIF-8, we fixed the usage of CHC to 16 mg, which is sufficient to inhibit the lactate fueled-respiration according to Figure 5A and 5B. Moreover, to get rid of the influence of varying the amounts of GOx, we used the mixture (8 mg) of active and denatured GOx (under different weight ratios) to synthesize CHC/GOx@ZIF-8. By these strategies, different CHC/GOx@ZIF-8 samples with similar morphology and loading amounts of CHC and GOx (including the active and denatured GOx) were obtained, in which the weight ratios between CHC and active GOx varied.

Cells cultured in media supplied with lactate (10 mM) and glucose (10 mM) as the dual carbon sources were incubated with the synthesized CHC/GOx@ZIF-8 (20 μg/mL) with the weight ratios between active GOx and CHC of 4:16, 5:16, 6:16, 7:16, and 8:16 for 24 hours, respectively. According to the CCK-8 analysis (Figure S6), the cell proliferation was significantly reduced as the weight ratios between active GOx and CHC increased. The optimized synergistic effect was observed at the weight ratio of 6:16, and the anti-tumor effect showed no further enhancement as the ratio increased. Therefore, in this work, 16 mg CHC and 6 mg GOx were used to synthesize CHC/GOx@ZIF-8 and conduct the following experiments.

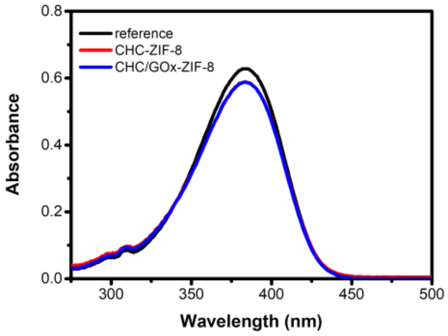

Figure S7. Absorbance spectra of CHC in the supernatants during the synthesis of CHC@ZIF-8 and CHC/GOx@ZIF-8.

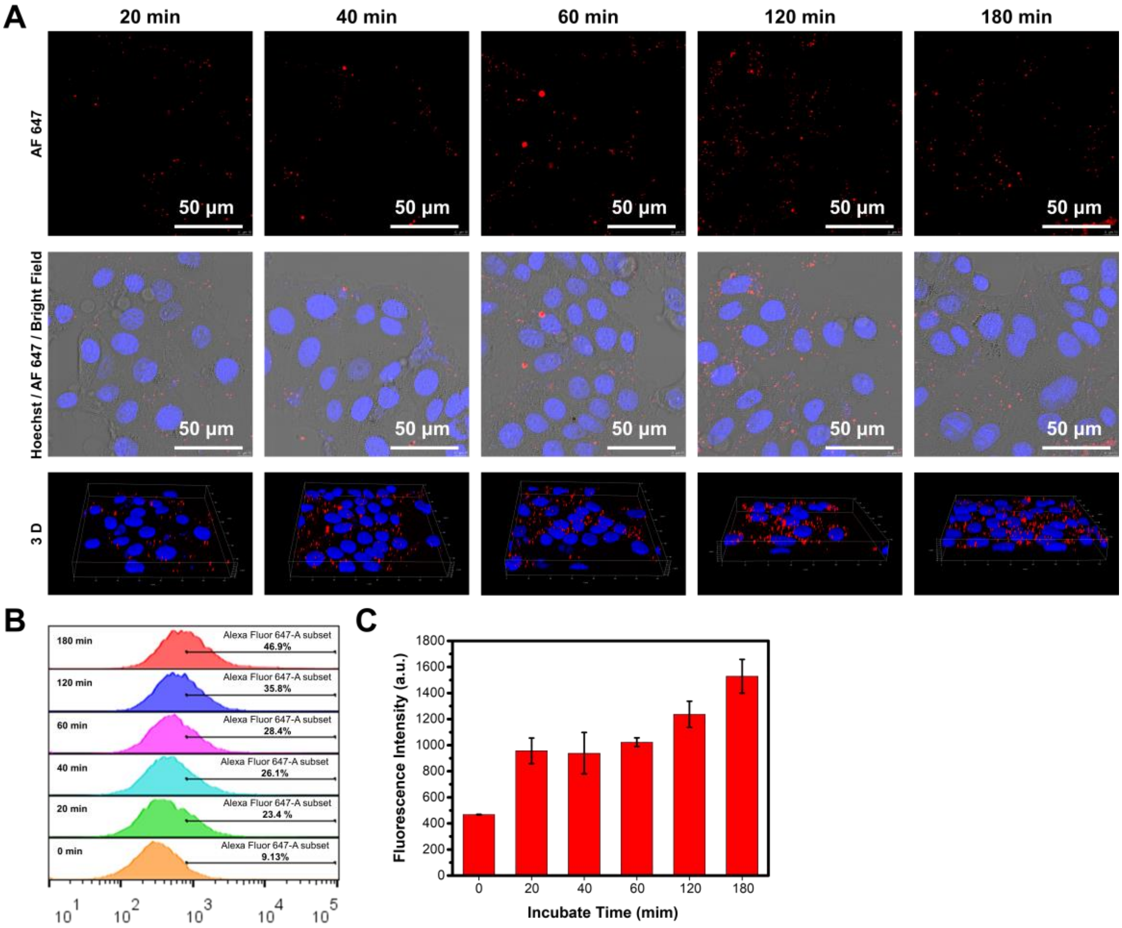

Figure S8. (A) LSCM and (B) FCM analysis of the cellular internalization of CHC/GOx@ZIF-8 (20 μg/mL). (C) Corresponding mean fluorescence intensity according to FCM analysis. Scale bar: 50 μm.

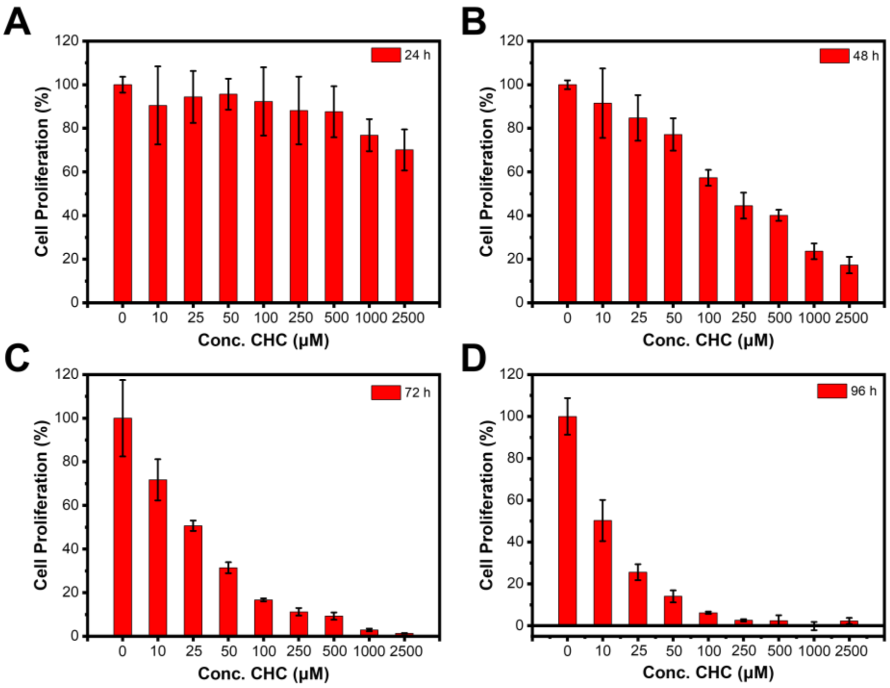

Figure S9. Cell proliferation after incubation with CHC in medium supplied with lactate (10 mM) for (A) 24, (B) 48, (C) 72 and (D) 96 hours. Data presented as mean ± SD, n=3. Cells with out any treatment was set as the control group and cell proliferation of the control group was set as 100%.

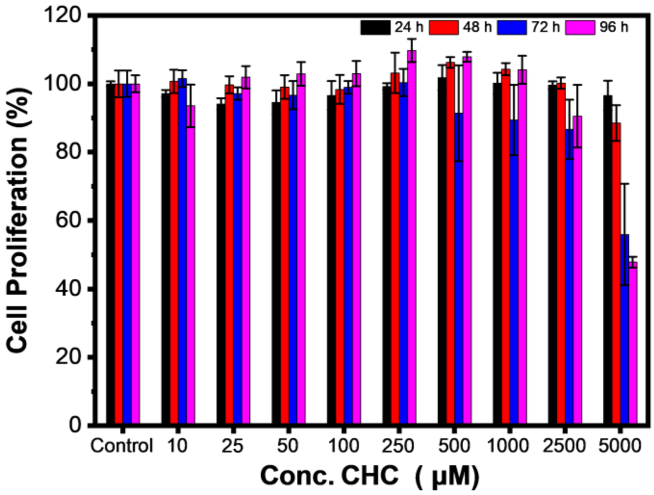

Figure S10. Cell proliferation after incubation with CHC in the medium supplied with glucose (10 mM) for different periods. Data presented as mean ± SD, n=3. Cells with out any treatment was set as the control group and cell proliferation of the control group was set as 100%.

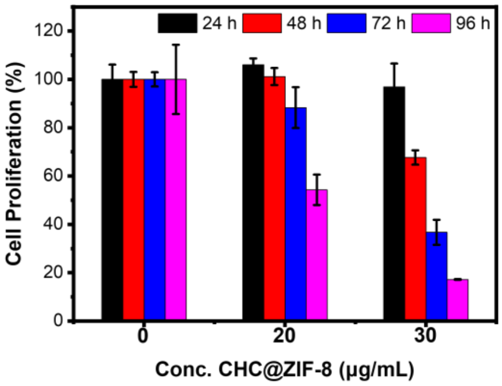

Figure S11. Cell proliferation after incubation with CHC@ZIF-8 in the medium supplied with lactate (10 mM) for different periods. Data presented as mean ± SD, n=3. Cells with out any treatment was set as the control group and cell proliferation of the control group was set as 100%.

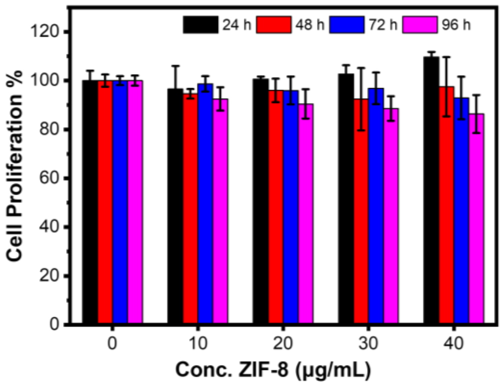

Figure S12. Cell proliferation after incubation with different concentrations of ZIF-8 for different periods in DMEM supplied with glucose (10 mM) and lactate (10 mM).

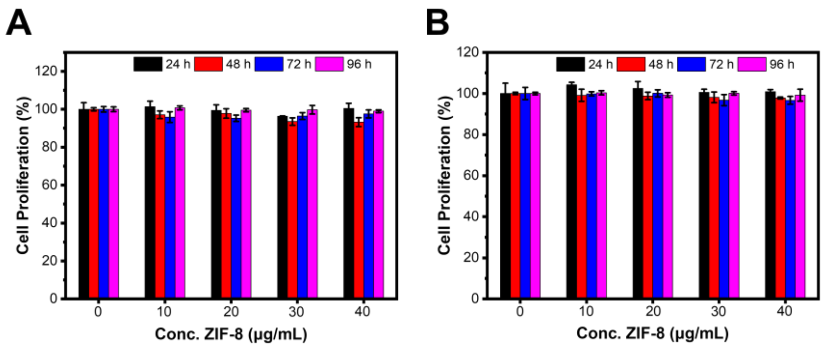

Figure S13. Cell proliferation after incubation with different concentrations of ZIF-8 for different periods in DMEM supplied with 10% FBS and using (A) glucose (10 mM) or (B) lactate (10 mM) as a single carbon source.

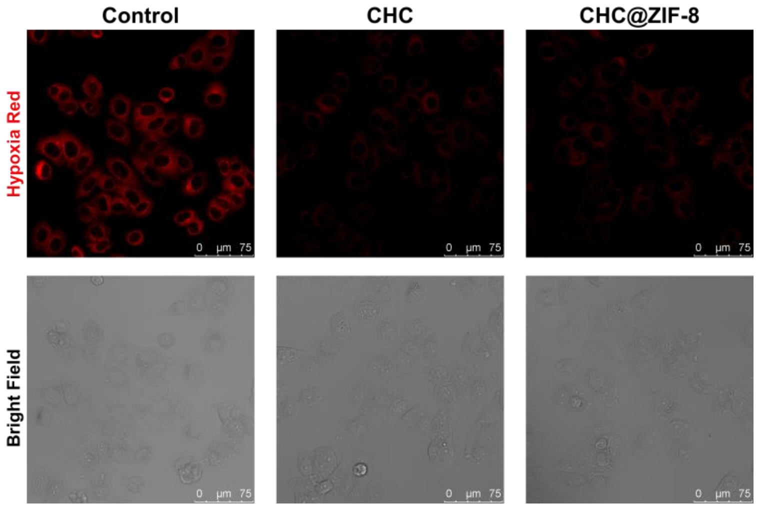

Figure S14. LSCM images of cells stained with Hypoxia Red after different treatment. Scale bar: 75 μm.

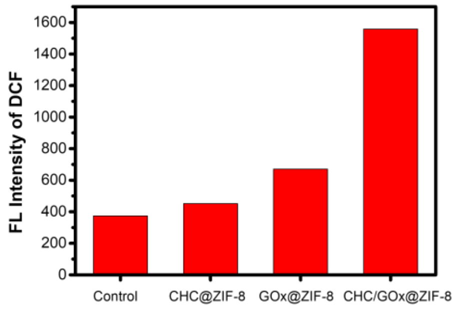

Figure S15. Mean fluorescence intensity of DCF according to the FCM analysis of cells after different treatments.

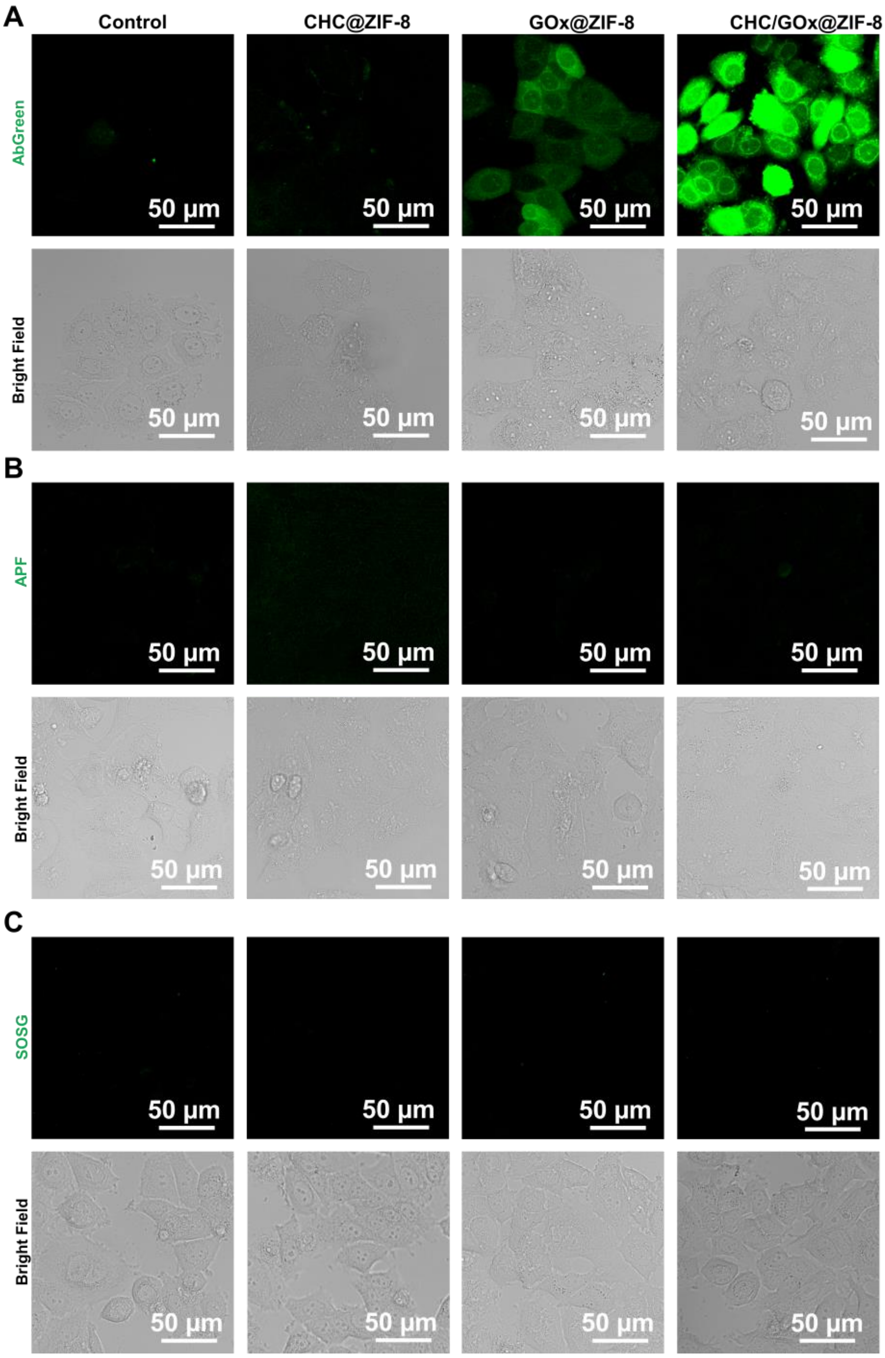

Figure S16. LSCM images of cells stained with (A) AbGreen, (B) APF and (C) SOSG after different treatment for the detection of H_2O_2, ·OH and $^1O_2$, respectively. Scale bar: 50 μm.

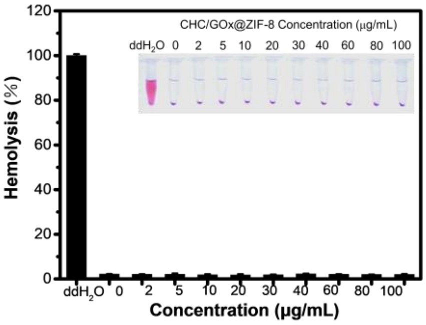

Figure S17. Hemolytic activities of CHC/GOx@ZIF-8 (2–100 μg/mL) suspensions toward RBCs in PBS. Hemolysis caused by double-distilled water (ddH_2O) and PBS were set as positive and negative control, respectively. The hemolysis activity of positive control was set as 100 %.

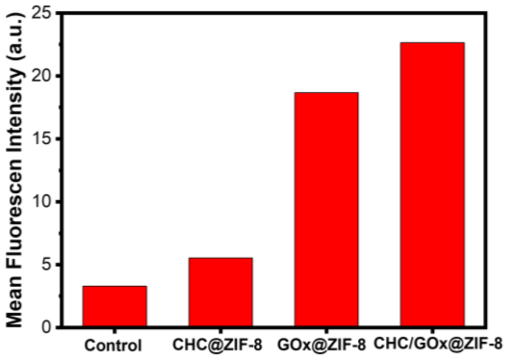

Figure S18. Quantitative fluorescence intensity of antibodies labeling the caspase-3 in Figure 6I using the Image J 2X software.

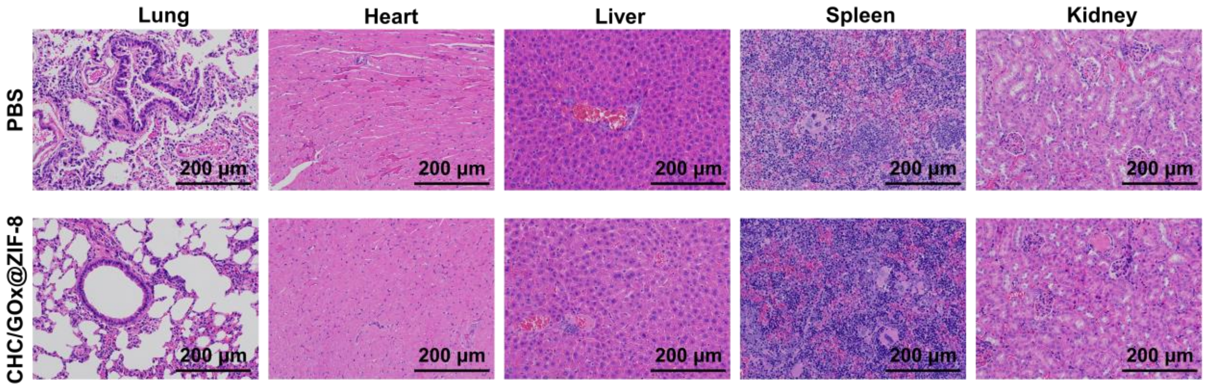

Figure S19. H & E staining of major organs of the mice 16 days after treatment with PBS and CHC/GOx@ZIF-8. All the images were achieved under the same magnification. Scale bar: 200 μm.
